# The Protective Effects of Osteocyte‐Derived Extracellular Vesicles Against Alzheimer's Disease Diminished with Aging

**DOI:** 10.1002/advs.202105316

**Published:** 2022-05-04

**Authors:** Ya‐Ling Jiang, Zhen‐Xing Wang, Xi‐Xi Liu, Mei‐Dan Wan, Yi‐Wei Liu, Bin Jiao, Xin‐Xin Liao, Zhong‐Wei Luo, Yi‐Yi Wang, Chun‐Gu Hong, Yi‐Juan Tan, Ling Weng, Ya‐Fang Zhou, Shan‐Shan Rao, Jia Cao, Zheng‐Zhao Liu, Teng‐Fei Wan, Yuan Zhu, Hui Xie, Lu Shen

**Affiliations:** ^1^ Department of Neurology Xiangya Hospital Central South University Changsha Hunan 410008 China; ^2^ Department of Orthopedics Movement System Injury and Repair Research Center Xiangya Hospital Central South University Changsha Hunan 410008 China; ^3^ National Clinical Research Center for Geriatric Disorders (Xiangya Hospital) Changsha Hunan 410008 China; ^4^ Engineering Research Center of Hunan Province in Cognitive Impairment Disorders Central South University Changsha Hunan 410008 China; ^5^ Hunan International Scientific and Technological Cooperation Base of Neurodegenerative and Neurogenetic Diseases Changsha Hunan 410008 China; ^6^ Key Laboratory of Hunan Province in Neurodegenerative Disorders Central South University Changsha Hunan 410008 China; ^7^ Department of Sports Medicine Xiangya Hospital Central South University Changsha Hunan 410008 China; ^8^ Xiangya Nursing School Central South University Changsha Hunan 410013 China

**Keywords:** aging, Alzheimer's disease, extracellular vesicles, osteocyte, osteoporosis

## Abstract

Both Alzheimer's disease (AD) and osteoporosis (OP) are common age‐associated degenerative diseases and are strongly correlated with clinical epidemiology. However, there is a lack of clear pathological relationship between the brain and bone in the current understanding. Here, it is found that young osteocyte, the most abundant cells in bone, secretes extracellular vesicles (OCY^Young^‐EVs) to ameliorate cognitive impairment and the pathogenesis of AD in APP/PS1 mice and model cells. These benefits of OCY^Young^‐EVs are diminished in aged osteocyte‐derived EVs (OCY^Aged^‐EVs). Based on the self‐constructed OCY‐EVs tracer transgenic mouse models and the in vivo fluorescent imaging system, OCY‐EVs have been observed to be transported to the brain under physiological and pathological conditions. In the hippocampal administration of A*β*40 induced young AD model mice, the intramedullary injection of *Rab27a*‐shRNA adenovirus inhibits OCY^Young^‐EVs secretion from bone and aggravates cognitive impairment. Proteomic quantitative analysis reveals that OCY^Young^‐EVs, compared to OCY^Aged^‐EVs, enrich multiple protective factors of AD pathway. The study uncovers the role of OCY‐EV as a regulator of brain health, suggesting a novel mechanism in bone‐brain communication.

## Graphical abstract

It is found that the osteocyte‐derived extracellular vesicles (OCY‐EVs) isolated from young osteocytes can ameliorate cognitive impairment and pathogenies of AD, but not OCY‐EVs isolated from aged osteocytes. OCY‐EV can transfer to the brain under physiological and pathological conditions. The study uncovers the role of OCY‐EVs as a regulator of brain, suggesting a novel mechanism in bone‐brain communication.

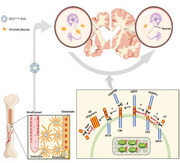

## 1 Introduction

Alzheimer's disease (AD) is a progressive neurodegenerative disease that is mainly characterized by a progressive decline in memory and cognitive function.^[^ ^1^ ^]^ The prevalent features of AD pathogenesis are the appearance of *β*‐amyloid (A*β*) plaques and neurofibrillary tangles, which cause microglial activation, synaptic deficiency, and neuronal loss.^[^ ^1^, ^2^ ^]^ During aging, increased A*β* formation and weakened A*β* cleavage capacity lead to toxic product accumulation, which crucially contributes to the development of AD.^[^ ^1^, ^3^ ^]^ Osteoporosis (OP) is a systemic skeletal disease characterized by low bone mass and microarchitectural deterioration of bone tissue, with a consequent increase in bone fragility and susceptibility to fracture.^[^ ^4^ ^]^ Skeletal aging leads to changes in the cellular function of bone tissue and an imbalance in bone remodeling between bone resorption and formation.^[^ ^5^ ^]^ Both AD and OP are common degenerative diseases related to aging and strongly correlate with clinical epidemiology.^[^ ^6^ ^]^ Several studies have demonstrated that elderly individuals with dementia present a lower bone mineral density (BMD) and a higher frequency of OP at the hip, and individuals with low BMD are also associated with an increased risk of AD.^[^ ^7^ ^]^ AD and OP also share various potential mechanisms, such as immunosuppression, lack of physical activity, low levels of vitamin D and K, and the presence of the apolipoprotein E (ApoE) 4 allele.^[^ ^6^, ^8^ ^]^ However, there is a lack of clear pathological relationship between AD and OP in the current understanding.

Osteocytes (OCYs) are the most abundant cells (almost 90–95%) in the bone and play a vital role in bone homeostasis.^[^ ^9^ ^]^ OCYs, similar to neurons, present abundant cilia and cytoplasmic processes (≈40–100 per cell) radiating through the mineralized matrix to connect with adjacent cells.^[^ ^10^ ^]^ Considering the vast numbers and network‐like structures of OCY, it is possible that OCYs work like analogous endocrine cells and release secretory factors to regulate bone and distant extraskeletal (nonbone) organs. OCYs have been discovered to secrete the receptor activator for nuclear factor‐*κ*B ligand (RANKL), fibroblast growth factor‐23 (FGF‐23), and leptin to regulate bone metabolism, kidney, and brain functions through the circulation, respectively.^[^ ^11^ ^]^

Recently, it has been recognized that extracellular vesicles (EVs) are important factors to mediate the exchange of intercellular biological signals.^[^ ^12^ ^]^ OCYs reside in the lacunae within the hard‐mineralized bone matrix; moreover, bone matrix vesicles are anchored within the matrix and serve as the initial mineralization site.^[^ ^13^ ^]^ These phenomena suggest that the anchored vesicles are probably osteocyte‐derived EVs (OCY‐EVs). The secreted OCY‐EVs are released from the bone matrix during the process of bone resorption or transferred to distant organs through the intraskeletal vasculature. For instance, we previously reported that OCY‐EVs accumulated in the prostate, affecting the development of benign prostatic hyperplasia.^[^ ^14^ ^]^ These reports, together with the evidence‐based clinical relevance of AD and OP, suggest that the exchange of signals in the bone‐brain axis is likely to be mediated by OCY‐EVs.

Herein, we confirmed the clinical relevance between bone and brain health. The transport of bone‐derived OCY‐EVs was observed in brain tissue using self‐constructed OCY‐EVs tracing mice. Then, we isolated and characterized OCY‐EVs from primary osteocyte derived from 2‐ or 16‐month‐old mice (OCY^Young^‐EVs or OCY^Aged^‐EVs). In vitro, we compared the effects of OCY^Young^‐EVs and OCY^Aged^‐EVs on A*β* pathology and apoptosis of nerve cells transfected with AD pathogenic genes. In vivo, we investigated whether OCY^Young^‐EVs could ameliorate cognitive impairment and the pathogenesis of AD in early or later stages of APP/PS1 AD model mice. Moreover, in A*β*40‐induced AD model mice, we tested whether the intramedullary injection of *Rab27a*‐shRNA adenovirus could inhibit EVs secretion from bone and aggravate cognitive impairment. Furthermore, potential protective factors for AD in OCY‐EVs were screened by proteomic quantitative analysis. This work showed that young bone‐derived OCY‐EVs played a protective role in AD through the bone‐brain axis.

## 2 Results

### 2.1 Bone Health Strongly Correlated with Brain Health in Clinical Epidemiology

**Figure 1:**
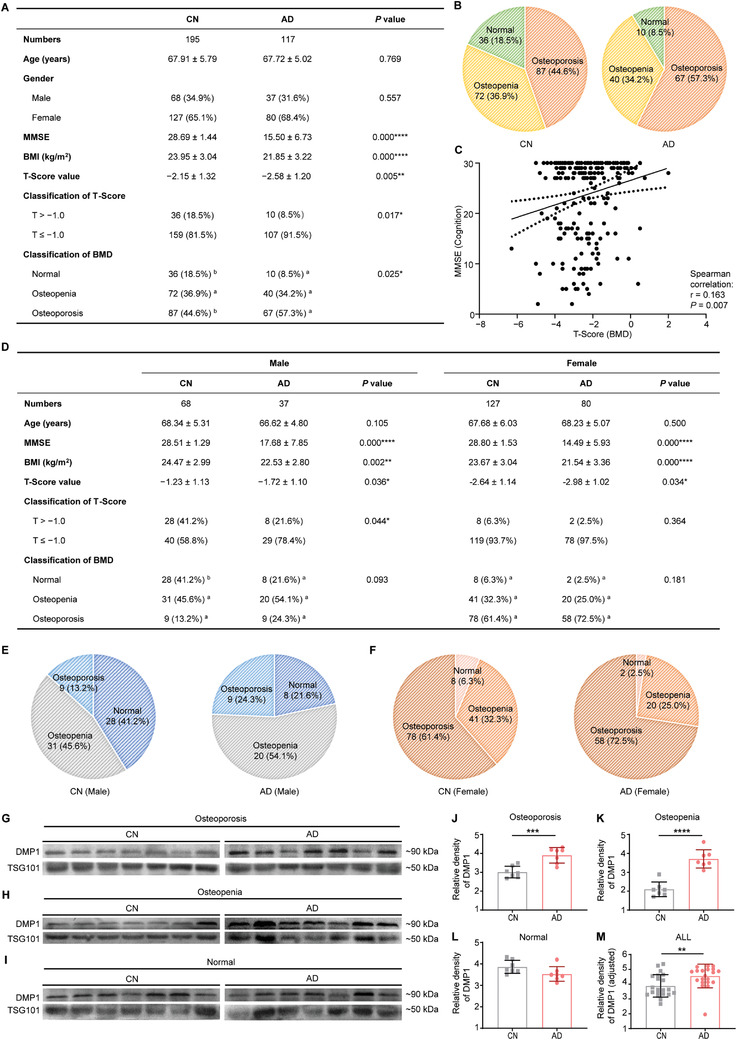
Bone Health Strongly Correlated with Brain Health in Clinical Epidemiology. A) Basic information and BMD of CN and AD subjects. a,b) each superscript letter denoted a subset of intervention categories whose column proportions did not differ significantly from each other at the 0.05 level (Bonferroni method). B) The distributions of three categories of BMD (Osteoporosis, Osteopenia, and Normal) in CN and AD subjects. C) Scatter plot of T‐Score and MMSE showing the relationship between BMD and cognition. D) Basic information and BMD of CN and AD subjects separated by gender. a,b) each superscript letter denoted a subset of intervention categories whose column proportions did not differ significantly from each other at the 0.05 level (Bonferroni method). The distributions of three categories of BMD (Osteoporosis, Osteopenia, and Normal) in E) male or F) female subjects. Representative western blot images of DMP1 and TSG101, and quantification of DMP1 intensity in plasma EVs from gender‐ and age‐matched CN subjects and AD patients with G,J) Osteoporosis, H,K) Osteopenia, or I,L) Normal. TSG101 was used as a marker of EVs. Each gel contained a calibration sample to adjust the comparison of two groups. *n* = 7 per group. M) Analysis of DMP1 intensity after correction for all samples in (G‐I). *n* = 21 per group. Data were presented as mean ± SD. For panel (A) and (D): Chi‐Square test for Gender, T‐Score classifications, and BMD classifications; unpaired, two‐tailed Student's *t*‐test for Age, MMSE, BMI, T‐Score value. For panel (C): Spearman Correlation Analysis. For panel (J‐L): unpaired, two‐tailed Student's *t*‐test. * *P* < 0.05, ** *P* < 0.01, *** *P* < 0.001, **** *P* < 0.0001.

To confirm the relationship between bone quality and cognitive impairment, bone mineral density (BMD) and Mini‐Mental State Examination (MMSE) tests were conducted in sex‐ and age‐matched subjects. The demographic and clinical characteristics of cognitively normal subjects (CN, *n* = 195) and AD patients (AD, *n* = 117) were summarized in **Figure** **1**A. The age and gender distribution of the two cohorts were matched (*P* > 0.05; Figure 1A). The mean MMSE score was 28.69 ± 1.44 in CN subjects and 15.50 ± 6.73 in AD patients (*P* = 0.000; Figure 1A), which was used to clinically estimate the cognitive impairment.^[^ ^15^ ^]^ According to World Health Organization (WHO) criteria, the bone density assessment was reported as T‐Scores.^[^ ^16^ ^]^ The T‐Score value of AD patients (−2.58 ± 1.20) presented markedly lower than the CN subjects (−2.15 ± 1.32, *P* = 0.005). In the clinic, a T‐Score > −1.0, −2.5 < T‐Score ≤ −1.0, or T‐Score ≤ −2.5 was referred to as normal, osteopenia, or osteoporosis, respectively.^[^ ^16^ ^]^ The frequency of bone loss (T‐Score ≤ −1.0) in AD patients was 91.5%, which was significantly higher than that in CN subjects (81.5%, *P* = 0.017; Figure 1A). The distributions of the three categories of BMD in CN subjects and AD patients were illustrated in Figure 1B. The percentage of AD patients with osteoporosis (57.3%) was higher than that of CN subjects (44.6%); nevertheless, the proportion of normal BMD in the AD (8.5%) and CN (18.5%) was reversed (*P* < 0.05; Figure 1A,B). Furthermore, there was a positive correlation between MMSE and T‐Score (Spearman correlation coefficient = 0.163, *P* = 0.007; Figure 1C).

Due to the incidence of AD and OP being different in male and female subjects, we then analyzed the data in a stratified manner by gender. The demographic and clinical characteristics of male and female subjects were summarized in Figure 1D. The BMI of AD patients (Male: 22.53 ± 2.80; Female: 21.54 ± 3.36) was significantly lower than that of CN subjects (Male: 24.47 ± 2.99, *P* = 0.002; Female: 23.67 ± 3.04, *P* = 0.000). The T‐Score value of AD patients (Male: −1.72 ± 1.10; Female: –2.98 ± 1.02) presented markedly lower than that of CN subjects (Male: −1.23 ± 1.13, *P* = 0.036; Female: −2.64 ± 1.14, *P* = 0.034). In male subjects, the frequency of bone loss (T‐Score ≤ −1.0) in AD patients was 78.4%, which was significantly higher than that in CN subjects (58.8%, *P* = 0.044; Figure 1D). However, in female subjects, the frequency of bone loss (T‐Score ≤ −1.0) in AD patients was slightly higher than CN subjects in trend (97.5% and 93.7% respectively, *P* = 0.364; Figure 1D). The distributions of the three categories of BMD of male and female subjects were illustrated in Figure 1E,F. The percentage of AD patients with osteoporosis (Male: 24.3%, Female: 72.5%) was higher than that of relevant CN subjects (Male: 13.2%, Female: 61.4%). These clinical epidemiologist studies suggested that individuals with lower T‐Score tended to exhibit poorer cognitive function.

Changes in bone matrix properties and osteocyte dysregulation occurred with aging.^[^ ^17^ ^]^ Accompanied by bone loss and bone remodeling, the bone matrix and contents were absorbed and released into the surroundings and circulation.^[^ ^18^ ^]^ Among the extracellular secretions, EVs usually participated in distant interorgan communication.^[^ ^12^, ^19^ ^]^ Thus, plasma EVs from gender‐ and age‐matched AD and CN subjects were isolated to compare the different compositions. Western blot was used to identify the EVs classical marker of Tumor Susceptibility Gene 101 (TSG101), and measure the amount of osteocyte‐specific protein Dentin Matrix Protein 1 (DMP1) in plasma EVs. As shown in Figure 1D–F and quantified in Figure 1G–I, AD patients with osteoporosis (Figure 1D,G) or osteopenia (Figure 1E,H) had higher levels of OCY‐EVs in plasma than the related controls (*n* = 7 per group). However, there was no difference in patients and controls with normal BMD (Figure 1F,I, *n* = 7 per group). Representative plasma EVs from osteoporosis, osteopenia, and normal BMD were also displayed in one gel to avoid operation error (Figure S1A,B, Supporting Information). Collectively, these results showed that patients with AD (*n* = 21) had higher levels of OCY‐EVs in plasma than the controls (*n* = 21; Figure 1J).

### 2.2 OCY‐EVs Were Transported to the Brain under Physiological and Pathological Conditions

**Figure 2:**
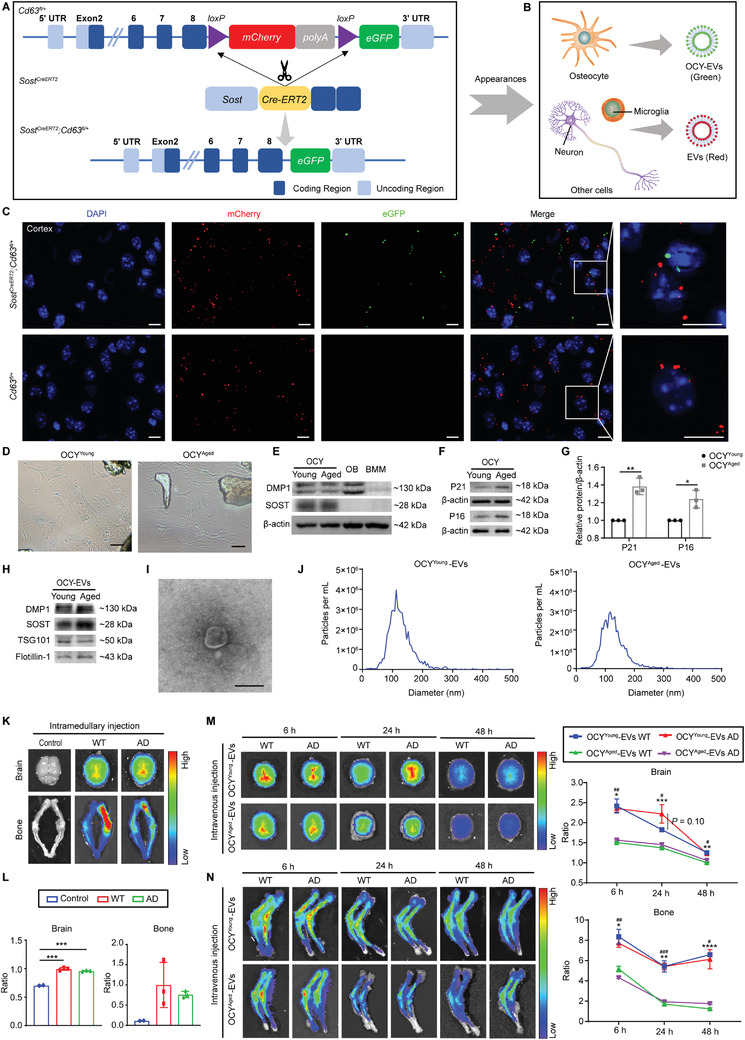
OCY‐EVs were Transported to the Brain under Physiological and Pathological Conditions. A) Schematic diagram of the construction of *Sost^CreERT2^;Cd63^fl/+^* mice model and B) its fluorescent appearances of EVs. C) Representative IF images of brain tissue sections of *Sost^CreERT2^;Cd63^fl/+^* mice and *Cd63^fl/+^* littermate control mice. DAPI (blue), mCherry (red), and eGFP (green). The merged photos were overlaid on the right side, and the white square indicated the amplification regions. Scale bar: 10 µm. Representative D) cellular morphologies, E) western blot images of DMP1 and SOST expression, F) western blot images and G) quantification of P21 and P16 expression in OCY^Young^ and OCY^Aged^. Scale bar: 100 µm. *β*‐actin was used as loading control. OB, Osteoblast; BMM, bone marrow macrophage. *n* = 3 per group. Representative H) western blot images of osteocyte markers (SOST and DMP1) and EVs classical markers (TSG101 and Flotillin‐1), I) morphological analysis, and J) diameter distribution of OCY^Young^‐EVs and OCY^Aged^‐EVs. Scale bar: 100 nm. K) Representative *ex vivo* fluorescent images and L) quantification of the fluorescent signals of brain and lower limb bone from WT and APP/PS1 AD mice treated with DiR‐labeled OCY‐EVs or solvent by intramedullary administration. *n* = 3 per group. Representative *ex vivo* fluorescent images and quantification of the fluorescent signals of M) brain and N) lower limb bone from WT and APP/PS1 AD mice treated with DIR‐labeled OCY^Young^‐EVs or DIR‐labeled OCY^Aged^‐EVs by intravenous injection. *n* = 3 per group, * OCY^Young^‐EVs WT (blue line) versus OCY^Aged^‐EVs WT (green line), # OCY^Young^‐EVs AD (red line) versus OCY^Aged^‐EVs AD (purple line). Data were presented as mean ± SD. For panel (G), (M), and (N): unpaired, two‐tailed Student's *t*‐test. For panel (L): one‐way ANOVA or the Kruskal–Wallis test with Bonferroni post hoc correction. */#*P* < 0.05, **/##*P* < 0.01, ***/###*P* < 0.001, ****/####*P* < 0.0001.

To verify the participation of OCY‐EVs in the communication between bone and brain, we first observed whether OCY‐EVs could be transported to the brain under physiological conditions. OCY‐EVs tracer transgenic mice were constructed by our group. The tetraspanin protein CD63 was enriched in the EVs membrane and considered as a ubiquitous marker for almost all EVs.^[^ ^20^ ^]^ To generate EV reporter mice (*Cd63^fl/+^* mice), the *loxp‐cc‐mCherry‐polyA‐loxp‐cc‐eGFP* expression frame was knocked in the termination codon in exon 8 of the *Cd63* gene by homologous recombination CRISPR/Cas9 technology (**Figure** **2**A). Before mating with Cre tool mice, the mCherry fusion protein was expressed. After mating with Cre tool mice, the eGFP fusion protein was conditionally expressed in specific cells. Sclerostin (SOST) was a well‐known specific marker of osteocytes.^[^ ^9^ ^]^ The *Sost* gene start codon site was knocked in *CreERT2‐Wpre‐pA* expression frame to construct *Sost^CreERT2^* mice, which expressing a tamoxifen‐activated Cre recombinase in osteocytes under the control of the sclerostin promoter (Figure 2A). The *Sost^CreERT2^* mice were mated with *Cd63^fl/+^* mice to obtain CD63 conditional mutagenic mice. The *Sost^CreERT2^;Cd63^fl/+^* mice (genotyping identified in Figure S2A,D, Supporting Information) should transcribe eGFP in osteocyte and OCY‐EVs, and mCherry in other cells and secreted EVs (such as neurons and microglia; Figure 2B). As shown in the cortex area of brain sections in Figure 2C, mCherry red fluorescence signals were observed in both *Sost^CreERT2^;Cd63^fl/+^*, and *Cd63^fl/+^* littermate control mice, while green fluorescence of eGFP was only observed in *Sost^CreERT2^;Cd63^fl/+^* mice. To further confirm these results, *Dmp1^Cre^* mice expressing Cre recombinase under the control of the murine Dmp1 promoter were used as an alternative tool to generate osteocyte specific targeted mutants.^[^ ^21^ ^]^ The obtained *Dmp1^Cre^;Cd63^fl/+^* mice (genotyping identified in Figure S2B,D, Supporting Information) showed similar eGFP and mCherry expression in the cortex area of brain sections (Figure S3A,B, Supporting Information). Although *Dmp1^Cre^* mice were widely used in osteocyte‐specific targeting, previous evidence had revealed that it also targeted cells in the mouse cerebellum and hindbrain.^[^ ^22^ ^]^ To prove the osteocytic source of these green fluorescence signals, the brain sections were also stained with SOST antibody. SOST‐positive staining mostly overlapped with the eGFP signals in *Dmp1^Cre^;Cd63^fl/+^* mice (Figure S3B, Supporting Information). These results suggest that OCY‐EVs (SOST^+^, DMP1^+^, and CD63‐eGFP^+^) could reach the brain under physiological conditions.

Next, we explored whether these OCY‐EVs could be transported from bone tissue to AD‐impaired brain tissue under pathological conditions by intramedullary injection of OCY‐EVs into APP/PS1 mice. Primary OCYs were derived from 2‐month‐old (OCY^Young^) and 16‐month‐old (OCY^Aged^) C57BL/6 mice. The biological features of osteocytes were characterized by cellular morphologies (Figure 2D), and DMP1 and SOST expression (Figure 2E).^[^ ^9^ ^]^ The expression of P21 and P16 proteins was measured to evaluate the senescence state of OCY (Figure 2F,G). Next, OCY‐EVs were isolated from the cell culture medium by ultracentrifugation and characterized with osteocyte markers (DMP1 and SOST) and EVs markers (TSG101 and Flotillin‐1) by western blot (Figure 2H). Moreover, transmission electron microscopy (TEM; Figure 2I) and nanoparticle tracking analysis (NTA; Figure 2J) results showed that OCY‐EVs displayed cup‐shaped morphologies with diameters of 127.1 ± 41.6 nm (OCY^Young^‐EVs) or 128.9 ± 45.8 nm (OCY^Aged^‐EVs), consistent with previous reports.^[^ ^12^ ^]^ To monitor the organ distributions, OCY^Young^‐EVs were labeled with DiR near‐infrared fluorescent dye and then intramedullary (the femoral bone marrow) administered into wild type (WT) or APP/PS1 AD mice (genotyping identified in Figure S2C,D, Supporting Information). After 24 h, *ex vivo* fluorescence imaging revealed strong fluorescence signals in the brain tissues, indicating that OCY‐EVs could enter the brains of WT and AD mice (Figure 2K,L). DiR fluorescent signals were also detected in the heart, liver, spleen, lung, and kidney (Figure S4A,B, Supporting Information). To further detect OCY‐EVs infiltration inside the tissues, red fluorescent dye DiL labeled OCY^Young^‐EVs (intramedullary administered) were also observed in tissue sections of bone, brain, and other organs of WT and AD mice by fluorescence microscopy (Figure S4C,D, Supporting Information).

Furthermore, to compare the different distribution dynamics of OCY^Young^‐EVs and OCY^Aged^‐EVs in WT and AD mice, *ex vivo* fluorescent imaging of the brain and lower limbs was performed at 6, 24, and 48 h after intravenous administration of DiR‐labeled OCY‐EVs. Compared with OCY^Aged^‐EVs, more OCY^Young^‐EVs entered the brain and bone in both WT and AD mice (Figure 2M,N) but not always in the other tissues (Figure S5A,B, Supporting Information). As shown in Figure 2M, the fluorescence signals of OCY^Young^‐EVs and OCY^Aged^‐EVs in the brain decreased over time in both WT and AD mice. However, the fluorescence signals of OCY^Young^‐EVs in the brain seemed to decline at a slower rate in AD mice than in WT mice (Figure 2M). Combined with the DiL intensity in the CA3 region of brain sections (Figure S6A,D, Supporting Information), it suggested that OCY^Young^‐EVs might persist longer in the brains of AD mice.

### 2.3 OCY^Young^‐EVs, but not OCY^Aged^‐EVs, Rescued the A*β* Pathology and Apoptosis of AD Model Cells

**Figure 3:**
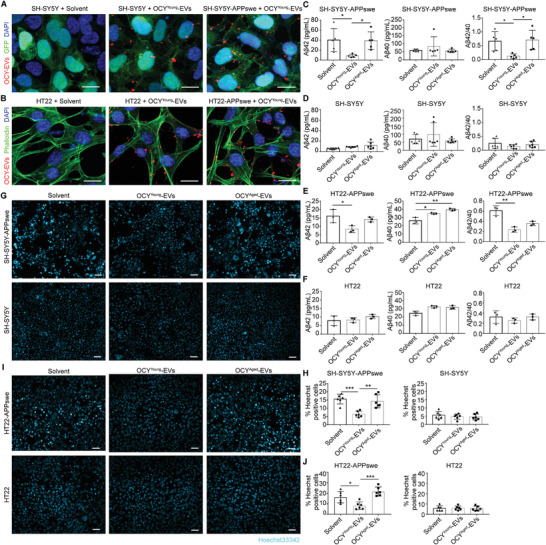
OCY^Young^‐EVs, but not OCY^Aged^‐EVs, Rescued the A*β* Pathology and Apoptosis of AD Model Cells. Internalization of the red fluorescent dye PKH26‐labeled OCY^Young^‐EV or solvent by A) SH‐SY5Y and SH‐SY5Y‐APPswe cells, B) HT22 and HT22‐APPswe cells. Scale bar: 20 µm. ELISA analysis the concentration and ratio of A*β*42 and A*β*40 of C) SH‐SY5Y‐APPswe, D) SH‐SY5Y, E) HT22‐APPswe, and F) HT22 cells treated with OCY^Young^‐EV, OCY^Aged^‐EV, or solvent. *n* = 5 in (C), *n* = 6 in (D), or *n* = 3 in (E‐F). Representative fluorescent images and quantification of Hoechest‐positive cells (light blue) of G, H) SH‐SY5Y‐APPswe and SH‐SY5Y, I, J) HT22‐APPswe and HT22 cells treated with OCY^Young^‐EV, OCY^Aged^‐EV, or solvent. *n* = 6 per group. Scale bar: 50 µm. Data were presented as mean ± SD. All dot plots: one‐way ANOVA or the Kruskal–Wallis test with Bonferroni post hoc correction. * *P* < 0.05, ** *P* < 0.01, *** *P* < 0.001, **** *P* < 0.0001.

We next asked whether OCY^Young^‐EVs and OCY^Aged^‐EVs were able to directly interact with neural cells. In vitro, the human neuroblastoma cell line SH‐SY5Y and the mouse hippocampal neuronal cell line HT22 were adopted as normal neural model cells.^[^ ^23^ ^]^ They were also transfected with vectors carrying the APPswe fragment to generate SH‐SY5Y‐APPswe and HT22‐APPswe AD model cells. SH‐SY5Y‐APPswe and HT22‐APPswe cells highly expressed amyloid protein precursor (APP) mutant proteins and generated more pathogenic proteins (Figure S7A–D, Supporting Information). The SH‐SY5Y and HT22 cells were transfected with control vectors. To evaluate the EVs internalized by AD model cells, OCY^Young^‐EVs were labeled with PKH26 red fluorescent cell linker dye and the excess dye was disposed by ultrafiltration. PKH26‐labeled OCY^Young^‐EVs were cocultured with SH‐SY5Y, SH‐SY5Y‐APPswe, HT22, or HT22‐APPswe cells for 12 h. After washing the free EVs, many red fluorescent signals were detected in the perinuclear region under confocal microscopy (**Figure** **3**A,B), which showed that OCY‐EVs could be internalized by these normal and AD model cells.

Excessive accumulation of *β*‐amyloid peptide (A*β*) as amyloid plaques and sequential neuronal cell apoptosis remained the primary neuropathologic criteria for AD.^[^ ^24^ ^]^ To assess the influence of OCY‐EVs on the process of AD pathology, extracellular A*β* accumulation and the apoptosis rate were detected in OCY^Young^‐EV‐ or OCY^Aged^‐EV‐treated SH‐SY5Y‐APPswe and HT22‐APPswe AD model cells. A*β* isoforms were composed of 39–43 amino acids, among which the 40‐residue A*β* isoform (A*β*40) was more abundant, and the 42‐residue A*β* isoform (A*β*42) was closely linked to the progression of AD.^[^ ^25^ ^]^ After treatment for 48 h, the concentrations of A*β*42 and A*β*40 in the medium supernatant of SH‐SY5Y‐APPswe, SH‐SY5Y, HT22‐APPswe, and HT22 cells were examined by enzyme‐linked immunosorbent assay (ELISA; Figure 3C–F). Compared with the solvent group, we observed a decreased concentration of A*β*42 and ratio of A*β*42/A*β*40 in the OCY^Young^‐EV‐treated SH‐SY5Y‐APPswe (Figure 3C) and HT22‐APPswe cells (Figure 3E). However, A*β*42 and A*β*40 secretion were not affected in the OCY^Aged^‐EVs groups. The SH‐SY5Y and HT22 cell groups presented a very low degree of A*β*42 accumulation, and no significant difference was observed after EVs intervention (Figure 3D,F). Finally, apoptotic neuronal cells were also detected by Hoechst staining. Compared with SH‐SY5Y and HT22 cells, SH‐SY5Y‐APPswe and HT22‐APPswe cells exhibited high levels of apoptosis (Hoechst‐positive cells), which were markedly rescued by OCY^Young^‐EVs (Figure 3G–J). Therefore, these results suggested that OCY^Young^‐EVs might play a protective role in the pathogenesis of AD (A*β* pathology and apoptosis).

### 2.4 OCY^Young^‐EVs, but not OCY^Aged^‐EVs, Ameliorated Cognitive Impairment and A*β* Pathology in the Early Stage of AD Model Mice

**Figure 4:**
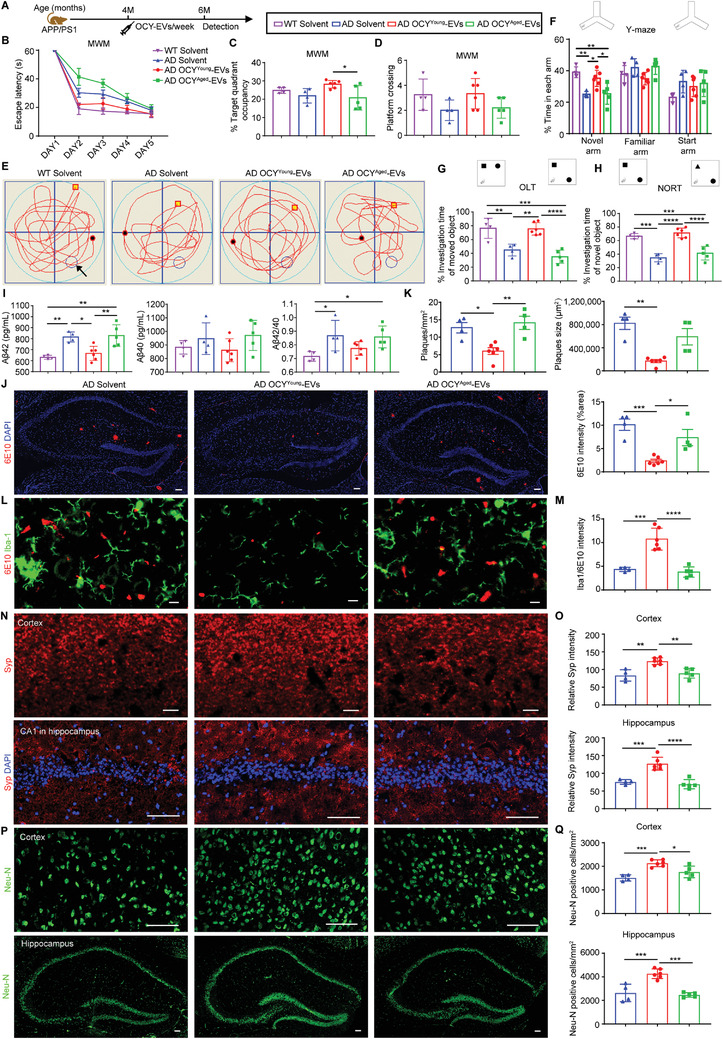
OCY^Young^‐EVs, but not OCY^Aged^‐EVs, Ameliorated Cognitive Impairment and A*β* Pathology in the Early Stage of AD Model Mice (4‐month‐old APP/PS1 mice). A) Experimental design. Treatment and detection time points were labeled. In the MWM test, B) the escape latency time, C) target quadrant occupancy, D) platform crossing times, and E) representative motion trajectories of different treated mice. The black arrow indicated the platform. F) Y‐Maze, G) OLT, and H) NORT performances of different treated mice. I) The levels and ratio of A*β*42 and A*β*40 in the brain tissue homogenate of different treated mice. J) Representative IF staining of 6E10 antibody (A*β*) and K) quantification of amyloid plaques per mm^2^ area, amyloid plaque size (µm^2^), and 6E10 staining intensity in the brain sections. Scale bar: 50 µm. L) Dual IF staining of Iba1 antibody (microglia) and 6E10 antibody (A*β*), and M) the ratio of Iba1/6E10 in the brain sections. Scale bar: 10 µm. Representative IF staining of N) synaptophysin (Syp) and P) mature neurons (Neu‐N), and quantification of O) relative Syp intensity and Q) Neu‐N positive cells per mm^2^ in the cortex and hippocampus of the brain section. Scale bar: 10 µm (N, Cortex), 50 µm (N, Hippocampus; P, Cortex and Hippocampus). Solvent‐treated WT group, *n* = 4; solvent‐treated APP/PS1 group, *n* = 4; OCY^Young^‐EV‐treated APP/PS1 group, *n* = 6; OCY^Aged^‐EV‐treated APP/PS1 group, *n* = 5. Data were presented as mean ± SD. All dot plots: one‐way ANOVA or the Kruskal–Wallis test with Bonferroni post hoc correction. * *P* < 0.05, ** *P* < 0.01, *** *P* < 0.001, **** *P* < 0.0001.

To investigate the effects of OCY^Young^‐EVs and OCY^Aged^‐EVs on the process of AD in vivo, 4‐month‐old WT or APP/PS1 mice were intravenously administered equal amounts (2.4 × 10^6^ particles per gram of body weight) of OCY^Young^‐EVs, OCY^Aged^‐EVs, or solvent once a week for eight weeks (**Figure** **4**A). Four‐month‐old APP/PS1 mice began to exhibit A*β* deposition in the hippocampus and thus were considered as early stage AD model mice.^[^ ^26^ ^]^ After treatment, the Morris water maze (MWM), Y‐Maze, object location task (OLT), and novel object recognition task (NORT) were used to evaluate cognitive functions. The MWM and Y‐Maze tests are most commonly used to assess hippocampus‐dependent spatial learning and memory function.^[^ ^27^ ^]^ As shown in Figure 4B–E, compared with the solvent‐ or OCY^Aged^‐EV‐treated group, the behavior performance of OCY^Young^‐EV‐treated APP/PS1 mice seemed to be improved in the escape latency time in the learning trials (Figure 4B, Day 2 and Day 3), the target quadrant occupancy (Figure 4C) and the platform crossing times (Figure 4D,E) in the following probe trials (Day 6). In the Y‐Maze test, the time of the treated mice spent in the start arm, familiar arm, and novel arm (previously blocked) was recorded. OCY^Young^‐EV‐treated APP/PS1 mice explored the novel arm significantly longer than the solvent‐ or OCY^Aged^‐EV‐treated group (Figure 4F). Furthermore, OLT and NORT tests were commonly used as effective behavioral tasks to exploit the innate preference for the novelty to reveal memory for previously encountered objects.^[^ ^28^ ^]^ After OCY^Young^‐EV intervention, the mice exhibited improved spatial learning (OLT test, which heavily relies on hippocampal activity; Figure 4G) and nonspatial learning of object identity (NORT test, which relies on multiple brain regions; Figure 4H). However, after OCY^Aged^‐EV treatment, the mice maintained a comparable investigation time of moved or novel objects as the APP/PS1 control mice (Figure 4G,H).

Since A*β* accumulation and induced nerve injury were the most recognized pathogenesis of AD and the dominant pathology in APP/PS1 mice, we subsequently investigated whether OCY^Young^‐EVs could play a role in A*β* elimination and neuroprotection. First, the levels of soluble A*β*42 and A*β*40 in the supernatant from brain tissue homogenates were measured by ELISA. The concentrations of A*β*42 and the ratios of A*β*42/A*β*40 were significantly decreased in the OCY^Young^‐EV‐treated group compared with the solvent‐ or OCY^Aged^‐EV‐treated group (Figure 4I). The residual insoluble A*β* was also detected with the 6E10 antibody (recognizing the first 16 amino acids of the A*β* sequence) by western blot and presented the same trend (Figure S8A,B, Supporting Information). Then, IF staining with 6E10 antibody was conducted to identify A*β* plaques in brain sections. Compared with solvent or OCY^Aged^‐EVs, OCY^Young^‐EVs treatment markedly reduced the number and size of A*β* plaques and 6E10 antibody staining intensity in the hippocampus (Figure 4J,K), suggesting that OCY^Young^‐EVs ameliorated A*β* pathology in AD model mice. In addition, microglial cells commonly tended to phagocytose loosely organized A*β* to form dense‐core A*β* plaques, which served as a protective mechanism to prevent the dissemination of toxic preplaque A*β* oligomers throughout the brain.^[^ ^29^ ^]^ Dual IF staining of 6E10 and ionized calcium‐binding adaptor molecule 1 (Iba1; microglial marker) was conducted in different treated groups (Figure 4L). Although fewer microglia around plaques were observed in the OCY^Young^‐EVs‐treated group (Figure S8C, Supporting Information), the ratio of Iba1/6E10 was higher than that in the solvent‐ or OCY^Aged^‐EVs‐treated groups (Figure 4M). As a result, OCY^Young^‐EV‐treated APP/PS1 mice revealed an increased fluorescence intensity of synaptophysin (Syp, Figure 4N,O) and a higher number of Neu‐N immunoreactive cells (Figure 4P,Q) in both the cortex and hippocampus, suggesting that synaptic deficits and neuronal survival were improved. Interestingly, consistent with the published phenomenon,^[^ ^30^ ^]^ APP/PS1 mice displayed a noteworthy decrease in trabecular bone mass, which was reversed by OCY^Young^‐EV treatment (Figure S8D,E, Supporting Information). Collectively, the results indicated that OCY^Young^‐EVs ameliorated the behavioral performance and pathogenesis of early stage APP/PS1 mice.

### 2.5 OCY^Young^‐EVs, but not OCY^Aged^‐EVs, Rescued Cognitive Impairment and A*β* Pathology in the Later Stage of AD Model Mice

**Figure 5:**
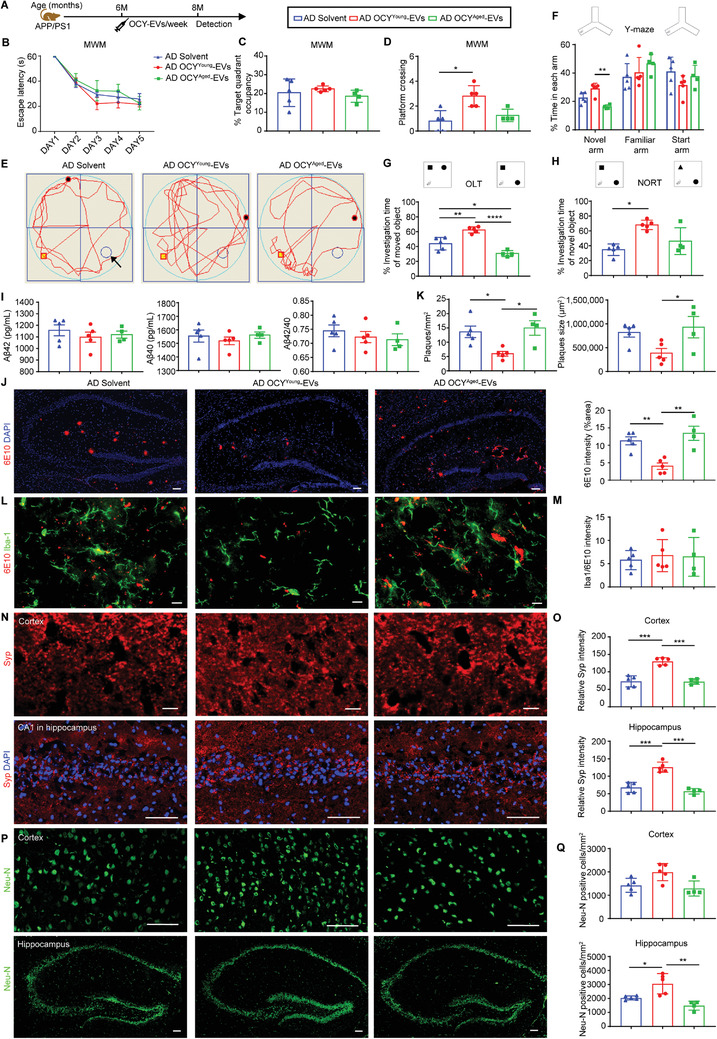
OCY^Young^‐EVs, but not OCY^Aged^‐EVs, Rescued Cognitive Impairment and A*β* Pathology in the Later Stage of AD Model Mice (6‐month‐old APP/PS1 mice). A) Experimental design. Treatment and detection time points were indicated. In the MWM test, B) the escape latency time, C) target quadrant occupancy, D) platform crossing times, and E) representative motion trajectories of different treated mice. The black arrow indicated the platform. F) Y‐Maze, G) OLT, and H) NORT performances of different treated mice. I) The levels and ratio of A*β*42 and A*β*40 in the brain tissue homogenate of different treated mice. J) Representative IF staining of 6E10 antibody (A*β*), and K) quantification of amyloid plaques per mm^2^ area, amyloid plaque size (µm^2^), and 6E10 staining intensity in the brain sections. Scale bar: 50 µm. L) Dual IF staining of Iba1 antibody (microglia) and 6E10 antibody (A*β*), and M) the ratio of Iba1/6E10 in the brain sections. Scale bar: 10 µm. Representative IF staining of N) synaptophysin (Syp) and P) mature neurons (Neu‐N), and quantification of O) relative Syp intensity and Q) Neu‐N positive cells per mm^2^ in the cortex and hippocampus of the brain section. Scale bar: 10 µm (N, Cortex), 50 µm (N, Hippocampus; P, Cortex and Hippocampus). Solvent‐treated APP/PS1 group, *n* = 5; OCY^Young^‐EV‐treated APP/PS1 group, *n* = 5; OCY^Aged^‐EV‐treated group, *n* = 4. Data were presented as mean ± SD. All dot plots: one‐way ANOVA or the Kruskal–Wallis test with Bonferroni post hoc correction. * *P* < 0.05, ** *P* < 0.01, *** *P* < 0.001, **** *P* < 0.0001.

APP/PS1 mice had been reported to exhibit cognitive impairment at six months of age;^[^ ^26^, ^31^ ^]^ thus, we further explored whether OCY^Young^‐EVs were able to reverse cognitive impairment and A*β* pathology. Six‐month‐old APP/PS1 mice were intravenously administered equal amounts (4.8 × 10^6^ particles per gram of body weight) of OCY^Young^‐EVs, OCY^Aged^‐EVs, or solvent weekly for eight weeks (**Figure** **5**A). In the OCY^Young^‐EVs intervention group, the escape latency time in the learning trials (Figure 5B) and the target quadrant occupancy in the subsequent probe trials (Figure 5C) of the MWM test, and the time spent in the novel arm of the Y‐Maze test (Figure 5F) tended to be better than those in the other groups. In particular, the platform crossing times in the probe trials of the MWM test were significantly greater than those of solvent‐ or OCY^Aged^‐EV‐treated mice (Figure 5D,E). Improved functional performance was also observed in OCY^Young^‐EV‐treated APP/PS1 mice in both the OLT and NORT tests (Figure 5G,H). Although the levels of A*β*42 or A*β*40 and the ratio of A*β*42/A*β*40 were comparable among the different groups (Figure 5I), insoluble A*β* was dramatically decreased in the OCY^Young^‐EV‐treated group compared with the solvent‐treated group (Figure S9A,B, Supporting Information). Pathologically, OCY^Young^‐EV treatment reduced the number of A*β* plaques and 6E10 antibody staining intensity in the brain sections of APP/PS1 mice (Figure 5J,K). Dual IF staining of Iba1 and 6E10 revealed a comparable ratio of Iba1/6E10 staining intensities in the OCY^Young^‐EV‐treated group (Figure 5L,M; Figure S9C, Supporting Information). An increase in Syp intensity (Figure 5N,O) and a higher number of Neu‐N immunoreactive cells (Figure 5P,Q) in the cortex and hippocampus were observed in OCY^Young^‐EV‐treated APP/PS1 mice. In contrast, the OCY^Aged^‐EV‐treated group showed no significant difference in vitro and in vivo compared to the solvent group. As expected, higher percent bone volume of trabecular bone (Tb. BV/TV) and trabecular number (Tb. N) levels were also observed in OCY^Young^‐EVs‐treated mice (Figure S9D,E, Supporting Information). Together, these findings indicated that OCY^Young^‐EVs rescued the behavioral performance and pathogenesis of later stage APP/PS1 mice.

### 2.6 Inhibition of EV Secretion from Bone Aggravated Cognitive Impairment in A*β*40‐induced Young AD Model Mice

**Figure 6:**
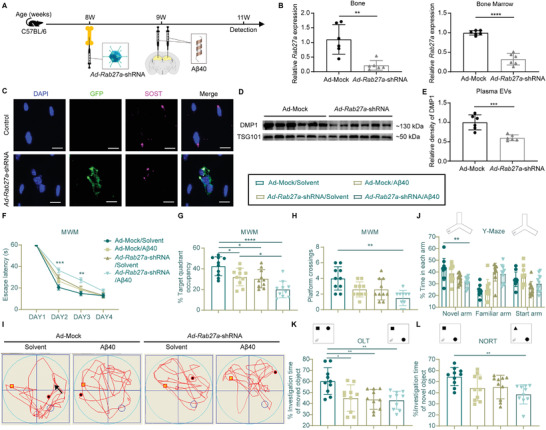
Inhibition of EV Secretion from Bone Aggravated Cognitive Impairment in A*β*40‐induced Young AD Model Mice. A) Experimental design. Treatment and detection time points were labeled. B) qRT‐PCR analysis of *Rab27a* mRNA expression levels in bone matrix and bone marrow of *Ad‐Rab27a*‐shRNA or Ad‐Mock treated mice. *n* = 6 per group. C) Representative IF staining of GFP (*Ad‐Rab27a*‐shRNA; green), SOST (OCY; purple), and DAPI (blue) in cortical bone samples from different treated mice. Scale bar: 10 µm. D) Representative western blot images and E) quantification of DMP1 intensity in plasma EVs from different treated mice. *n* = 6 per group. In the MWM test, F) the escape latency time, G) target quadrant occupancy, H) platform crossing times, and I) representative motion trajectories of different treated mice. The black arrow indicated the platform. J) Y‐Maze, K) OLT, and L) NORT performances of different treated mice. *n* = 10 per group. Data were presented as mean ± SD. For panel (B) and (E): unpaired, two‐tailed Student's *t*‐test. For panel (F), (G), (H), (J), (K), and (L): one‐way ANOVA or the Kruskal–Wallis test with Bonferroni post hoc correction. * *P* < 0.05, ** *P* < 0.01, *** *P* < 0.001, **** *P* < 0.0001.

The above results suggested that the isolated OCY^Young^‐EVs improved the impaired brains of APP/PS1 mice. These outcomes prompted us to study the features of physiologically secreted OCY^Young^‐EVs in vivo. In young mice, OCY^Young^‐EVs were constantly secreted and transported. Rab27a was generally perceived as a regulator of EV biogenesis and secretion.^[^ ^32^ ^]^ Thus, the adenoviral entrapped *Rab27a* shRNA silencing vector system (Ad‐*Rab27a*‐shRNA) and the control vector (Ad‐Mock) were constructed in this study. Eight‐week‐old WT mice were subjected to bilateral intramedullary injection of *Ad‐Rab27a*‐shRNA to inhibit bone‐derived EV secretion (**Figure** **6**A). As shown in Figure 6B, decreased expression of *Rab27a* was confirmed in the bone matrix and bone marrow following *Ad‐Rab27a*‐shRNA administration. IF staining of bone sections indicated that the intramedullary injected adenovirus (characterized with GFP, green) could infect the OCYs (characterized with SOST, purple; Figure 6C). As a result, markedly reduced OCY‐EVs were detected in the plasma EVs of *Ad‐Rab27a*‐shRNA‐treated mice (Figure 6D,E).

After one week, a certain amount of A*β*40 or solvent was administered into the bilateral hippocampus to mimic A*β* accumulation in this brain region using a high‐precision brain stereotaxic method (Figure 6A; Figure S10, Supporting Information). Two weeks later, cognitive functions were assessed in Ad‐Mock/Solvent, Ad‐Mock/A*β*40, *Ad‐Rab27a*‐shRNA/Solvent, or *Ad‐Rab27a*‐shRNA/A*β*40 mice. In the MWM and Y‐Maze tests, spatial learning and memory were slightly impaired in the Ad‐Mock/A*β*40 and *Ad‐Rab27a*‐shRNA/Solvent groups (Figure 6F–J). The worst cognitive functional performance was observed in the *Ad‐Rab27a*‐shRNA/A*β*40 group, as determined by the longest escape latency time in the learning trials (Figure 6F, Day 2 and Day 3), the least target quadrant occupancy in the following probe trials (Figure 6G), the least platform crossing times (Figure 6H,I), and the shortest exploring time in the novel arm (Figure 6J). Furthermore, *Ad‐Rab27a*‐shRNA/A*β*40‐treated mice exhibited significantly decreased investigation times of moved or novel objects in the OLT and NORT tests (Figure 6K,L). Collectively, these results suggested that inhibition of OCY^Young^‐EVs could accelerate severe cognitive impairment in young mice.

### 2.7 Multiple Functional Factors of A*β* Degradation and Mitochondrial Energy Metabolism were Enriched in OCY^Young^‐EVs

**Figure 7:**
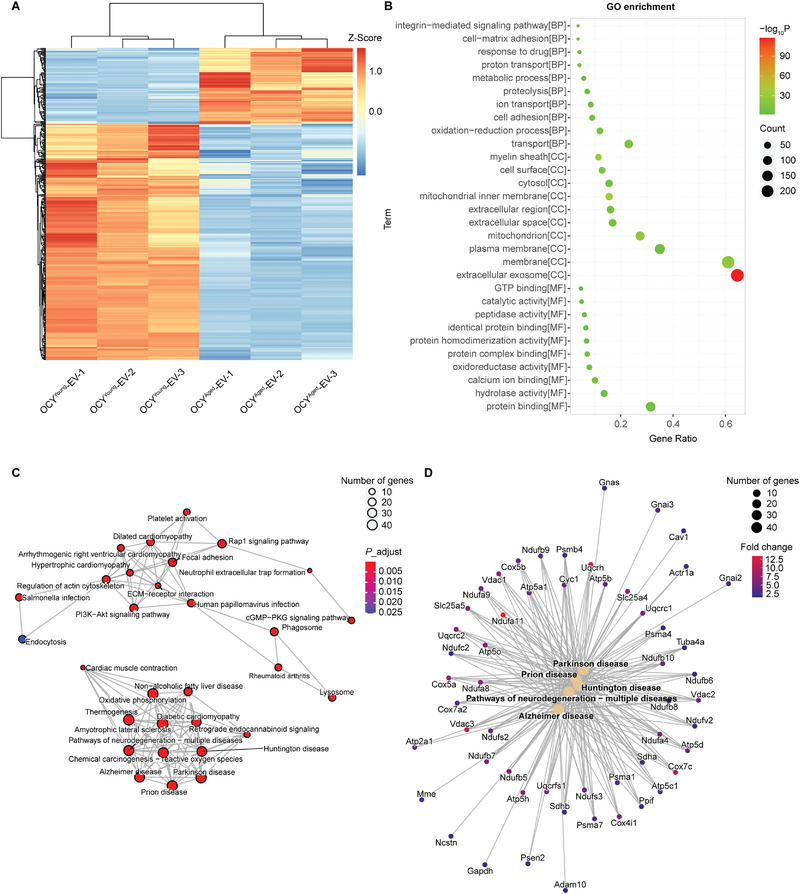
Multiple Functional Factors of A*β* Degradation and Mitochondrial Energy Metabolism were Enriched in OCY^Young^‐EVs. A) Heatmap of the differentially enriched proteins between OCY^Young^‐EVs and OCY^Aged^‐EVs (more than 1.5‐fold, *P* < 0.05). The protein levels were normalized by Z‐Score. B) The top 30 enriched GO terms (BP, CC, and MF) of differentially expressed proteins between OCY^Young^‐EVs and OCY^Aged^‐EVs (OCY^Young^‐EVs/OCY^Aged^‐EVs > 1.5). C) The network of top 30 enriched KEGG pathway terms of differentially expressed proteins (OCY^Young^‐EVs/OCY^Aged^‐EVs > 1.5). D) The network between enriched KEGG pathway (top 5) and genes. The fold change was used to show the ratio of protein expression levels between OCY^Young^‐EVs and OCY^Aged^‐EVs.

Furthermore, to explore the mechanism of the protective role of OCY^Young^‐EVs in AD pathway, global proteomics quantitative analysis of OCY^Young^‐EVs and OCY^Aged^‐EVs was performed using TMT labeling and LC–MS/MS. In total, 410 differently enriched proteins (higher than 1.5 times, *P* < 0.05) were identified between OCY^Young^‐EVs and OCY^Aged^‐EVs (Table S1, Supporting Information). Among these proteins, 310 and 100 proteins were highly expressed in OCY^Young^‐EVs and OCY^Aged^‐EVs, respectively (**Figure** **7**A). GO analysis was conducted to explore the biological interpretation and the involved biological processes. According to the functional enrichment results, 135 biological process (BP) terms, 104 cell component (CC) terms, and 83 molecular function (MF) terms were significantly enriched among the differentially expressed proteins (OCY^Young^‐EVs/OCY^Aged^‐EVs > 1.5; Figure 7B). The most enriched terms were membrane and extracellular exosome terms, suggesting that OCY^Young^‐EVs contained more membrane proteins and exosomal proteins (Figure 7B). Next, KEGG pathway enrichment analysis was performed to identify the most significantly enriched pathways (Figure 7C). Interestingly, most of them were related to neurodegenerative diseases. The top five enriched pathways were those involved in AD, Parkinson's disease (PD), Pathways of neurodegeneration‐multiple diseases, Prion disease, and Huntington's disease (Figure 7D). In the AD pathway, the highly enriched proteins could be roughly divided into two categories: A*β* degradation (4 proteins) and mitochondrial energy metabolism (38 proteins) (Figure 7D). The *Mme* gene encoded neprilysin (NEP: 3.30‐fold) could fully degrade A*β* peptides.^[^ ^33^ ^]^ Nicastrin (NCSTN; 1.92‐fold) and Presenilin‐2 (PSEN2; 1.69‐fold), as components of *β*‐secretase, could continuously cleave the *α*‐secretase cleavage product and eventually form nontoxic products. Disintegrin and metalloproteinase domain‐containing protein 10 (ADAM10; 1.54‐fold), as the main component of *α*‐secretase, could regulate the alternative APP processing to prevent A*β* formation.^[^ ^34^ ^]^ They worked together to reduce the production and promote the degradation of A*β*. In addition, mitochondrial dysfunction was a fundamental pathological feature of AD because the accumulation of damaged neuronal mitochondria had been found in both clinical AD cases and in AD mouse models.^[^ ^35^ ^]^ The higher ratio of mitochondrial proteins in OCY^Young^‐EVs might help to provide extra energy for neurological function. In summary, the highly enriched A*β* degradation and functional factors of mitochondrial energy metabolism in OCY^Young^‐EVs synergistically prevented A*β* deposition and neuronal damage.

## 3 Discussion

**Figure 8:**
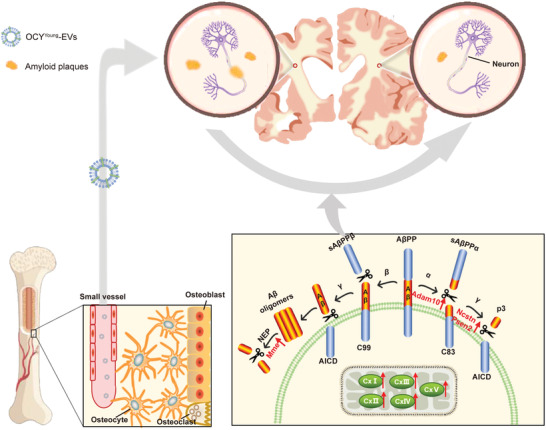
Schematic diagram. OCY‐EVs as the significant bone source messengers rescued brain lesions by transferring protective factors in the bone‐brain axis.

In our study, we present a novel mechanism underlying the correlation of OP and AD (**Figure** **8**). Two significant findings involve the bone‐brain axis. First, signal exchange between bone and brain can be achieved by transferring functional EVs from osteocytes in the bone to cells in the brain. Second, OCY^Young^‐EVs play a protective role in the AD pathway, while OCY^Aged^‐EVs lose this function. The effects of OCY‐EVs during aging have been demonstrated to change due to the different sorting of EVs contents.

Among the clinical subjects in our study, the BMD of AD patients is lower than that of sex‐ and age‐matched CN subjects, which is consistent with previous studies.^[^ ^7^ ^]^ Cognitive function is positively correlated with bone density. However, a high prevalence of OP was observed in female subjects both in CN and AD groups, and there was no significant difference existed in comparison of either BMD or T‐Score classification between CN and AD. The average age of menopausal is around 51–52 years old,^[^ ^36^ ^]^ and all women aged 65 years or older should be screened for osteoporosis as estrogen deficiency in postmenopausal women results in an increased prevalence of OP.^[^ ^37^ ^]^ Since the average age of female subjects in our cohort was 67.68 years in the CN group and 68.23 years in AD patients, loss of estrogen protection became the major cause of OP. More younger female individuals, especially between 40 to 60 years old, should be collected to reanalyze the difference in future research work.

Further exploration of plasma EVs has revealed that AD patients present more OCY‐EVs in plasma than CN subjects. This phenomenon may be due to the body compensation mechanism. The osteocytes try to release more OCY‐EVs to help the affected brain cells; unfortunately, OCY‐EVs derived from the elderly subjects lost their protective functions. Moreover, the OCY‐EVs tracer transgenic *Sost^CreERT2^;Cd63^fl/+^* and *Dmp1^Cre^;Cd63^fl/+^* mice have demonstrated that OCY‐EVs could reach the brain under physiological conditions. This connection under pathological condition is evaluated by the intramedullary injection of DiR‐labeled OCY‐EVs in APP/PS1 AD mice, which confirms that OCY‐EVs can be transferred from bone to the brain. The metabolic kinetics curve of OCY‐EVs in the brain indicates that more OCY^Young^‐EVs enter the brain than OCY^Aged^‐EVs, and OCY^Young^‐EVs decline at a slower rate in the brains of APP/PS1 AD mice than in those of WT mice. Proteomic analysis data showed that OCY^Young^‐EVs contain higher amounts of membrane and extracellular exosome proteins. These results probably explain why OCY^Young^‐EVs more easily penetrate the blood‐brain barrier than OCY^Aged^‐EVs.

Subsequently, we find that OCY^Young^‐EVs play a protective role in the pathogenesis of AD in vitro and in vivo. Decreased concentrations of A*β*42 and percentages of apoptotic cells are observed in OCY^Young^‐EV‐treated SH‐SY5Y‐APPswe and HT22‐APPswe cells. After intervention with OCY^Young^‐EVs, both early stage (4‐month‐old) and later stage (6‐month‐old) APP/PS1 AD model mice exhibit improved cognitive functions and fewer A*β* plaques, more synapses, and more neurons in the brain. However, the OCY^Aged^‐EV‐treated group shows no significant difference in vitro and in vivo, suggesting that the protective effects of OCY‐EVs decline with age. In later stage AD mice, the intervention amount of OCY‐EVs is increased to a double dose, the protective effect of OCY^Young^‐EVs is not as significant as the intervention in early stage AD mice. In OCY^Young^‐EVs treated AD mice, although the benefits of OCY^Young^‐EVs are observed in most of behavior and pathological tests, the small number of mice per group is a limitation in this study. Moreover, *Ad‐Rab27a*‐shRNA is designed to prevent the secretion of EVs. A*β*40 is used to induce young AD mice in 8‐week‐old WT mice. After intramedullary treatment with *Ad‐Rab27a*‐shRNA, plasma OCY^Young^‐EVs are reduced and the cognitive impairment of treated mice is aggravated. These results prove the effects of OCY^Young^‐EVs in preserving cognitive functions. Previous studies have reported that mesenchymal stem cell‐derived extracellular vesicles (MSC‐EVs) exhibit neuroprotective potential in AD.^[^ ^38^ ^]^ Intramedullary injection of *Ad‐Rab27a* shRNA could not only reach osteocytes but also block the secretion of EVs in other bone cells. The lack of a specific OCY‐EVs blocking tool is not addressed in this study.

Furthermore, the potential molecular mechanism of OCY^Young^‐EVs on neuroprotection is explored by proteomics analysis. KEGG pathway analysis of differentially enriched proteins (OCY^Young^‐EVs/OCY^Aged^‐EVs > 1.5) show that neurodegenerative diseases pathways are primarily enriched. In highly enriched proteins of the AD pathway, the functions of upregulated proteins include reducing A*β* production (such as ADAM10, NCSTN, and PSEN2), increasing A*β* degradation (such as NEP), and preserving extra‐mitochondrial.

In conclusion, our findings support that OCY^Young^‐EVs serve as important bone source messengers to preserve brain function in the bone‐brain axis (Figure 8).

## 4 Experimental Section

### Subjects

A total of 195 cognitive normal control subjects (67.91 ± 5.79 years old) and 117 AD patients (67.72 ± 5.02 years old) were involved in the study (Figure 1A). All patients were diagnosed by at least two experienced doctors from Xiangya Hospital according to the National Institute of Aging and Alzheimer's Association (NIA‐AA).^[^ ^39^ ^]^ Cognitive normal controls were recruited from the physical examination center of Xiangya Hospital. In addition, a Mini‐Mental State Examination (MMSE) ≥ 27 was administered as a standard for cognitive normal individuals.^[^ ^15^, ^40^ ^]^ All participants underwent dual‐energy X‐ray absorptiometry (DXA) measurement of the lumbar spine and total hip in the geriatric outpatient department of Xiangya Hospital. According to WHO criteria, BMD classifications of osteoporosis, osteopenia, and normal were defined as T‐Score ≤ −2.5, −2.5 < T‐Score ≤ −1.0, and T‐score > −1.0, respectively.^[^ ^16^ ^]^ The study was approved by the Ethics Committee of Xiangya Hospital, Central South University (institutional review board equivalent) (2019030501). Written informed consent was obtained from all participants involved in the study.

### Plasma Collection and Plasma EVs Extraction from Participants

Venous blood samples were collected in EDTA‐coated tubes using standard venipuncture protocols after an overnight fast. Then plasma was collected by centrifugation at 1000 rpm for 10 min at 4 °C. Whole plasma EVs were extracted by the ultracentrifugation method. Briefly, plasma was sequentially centrifuged at 2000 × *g* and 10 000 × *g* for 30 min to remove dead cells and debris. The supernatant was used to concentrate EVs by ultracentrifugation at 100 000 × *g* for 2 h at 4 °C. The EVs pellets were resuspended in PBS and stored at −80 °C until use.

### Primary OCY and OCY‐EVs Isolation

Primary OCY^Young^ or OCY^Aged^ were derived from 2‐ or 16‐month‐old C57BL/6 male mice according to the published protocol^[^ ^41^ ^]^ with slight modifications. In detail, the femora, tibiae, and humeri were aseptically dissected from the mice, and the remaining muscle, tendon, and periosteum were removed in *α*‐MEM (Cat. No. SH30265.01; HyClone, Logan, USA) with 2% penicillin and streptomycin (PS; Cat. No. P1400; Solarbio, Beijing, China). The bone epiphyses were cut off, and the bone marrow was flushed out. The bone was then cut into 1–2 mm lengths and washed in *α*‐MEM with 2% PS. Next, the bone pieces were incubated in warmed *α*‐MEM containing 10 active units per mL of collagenase II (Cat. No. 17 101 015; Gibco, Grand Island, USA) for 30 min at 37 °C, and then washed three times with phosphate‐buffered saline (PBS). The incubation step was repeated for another two times. Subsequently, the bone pieces were incubated in warmed 5 × 10^−3^ m ethylenediaminetetraacetic acid (EDTA; Cat. No. E809068; Macklin, Shanghai, China) solution at pH = 7.4 for 30 min and then washed three times with PBS. The alternate incubation of bone pieces in collagenase II and EDTA was conducted for another two times. Finally, the obtained bone particles were incubated on plates, which were precoated with 4% collagen I (Cat. No. 354 236; Corning, New York, USA) in 0.02 × 10^−6^ m acetic acid for one hour. *α*‐MEM with 5% fetal bovine serum (FBS; Cat. No. 12664‐025; Gibco), 5% calf serum (CS; Cat. No. A3520502; Gibco), and 2% PS was used as primary osteocyte culture medium. The plates were kept at 37 °C and 5% CO_2_ for seven days until the OCY crawled out from the bone slices. In addition, extractions of primary osteoblasts and bone marrow macrophages were performed according to the published protocols.^[^ ^42^ ^]^

To collect OCY‐EVs, the culture medium was changed to *α*‐MEM with 10% exosome depleted FBS (Cat. No. EXO‐FBS‐50A‐1; System Biosciences, Palo Alto, USA) and 1% PS. The cell culture medium was sequentially centrifuged at 300 × *g* for 10 min, 2000 × *g* for 30 min, and 10 000 × *g* for 30 min at 4 °C, and then the final supernatant was filtered through a 0.22‐µm filter (Millipore, Billerica, USA) to remove the residual cell debris. EVs were then pelleted by ultracentrifugation at 100 000 × *g* and 4 °C for 10 h in a Beckman Optima XPN ultracentrifuge (SW 32 Ti rotor; k‐factor 204). The EVs pellets were resuspended in PBS and stored at −80 °C until use (avoiding multiple freeze‐thaw cycles). The protein contents of OCY‐EVs were assessed by bicinchoninic acid (BCA) protein assay kit (Cat. No. 70‐PQ0012; MULTI SCIENCES, Hangzhou, China). The numbers and sizes of OCY‐EVs were tested by nanoparticle tracking analysis (NTA) using ZetaView PMX 110 (Particle Metrix, Meerbusch, Germany). The morphologies of OCY‐EVs were detected using a Hitachi H‐7650 transmission electron microscope (Hitachi, Tokyo, Japan). The efficacy and stability of each batch of OCY‐EVs were determined by cellular functional test and NTA analysis, respectively.

### In Vivo Distribution of OCY‐EVs

The OCY^Young^‐EVs and OCY^Aged^‐EVs were labeled with the lipophilic dye DiR iodide (Cat. No. 40757ES25; Yeasen, Shanghai, China) according to the manufacturer's instructions for *ex vivo* fluorescence imaging. After incubation, the solution mixture was transferred to Amicon Ultra‐0.5 Centrifugal filter units (10 kDa; Millipore) and centrifuged at 3000 × *g* to remove possible excess fluorescent dye. To investigate whether OCY‐EVs could reach the brain, DiR‐labeled OCY^Young^‐EVs were resuspended in PBS and injected in the intramedullary region (6 × 10^7^ particles in 10 µL PBS) of each mouse (4‐month‐old APP/PS1 mouse, *n* = 3). The mice in the control group (*n* = 3) were treated with an equal volume of solvent. Twenty‐four hours later, all mice were sacrificed to collect the brain, heart, liver, kidneys, femora, and tibiae. Fluorescent signals in these tissues were detected immediately and quantified with a fluorescence tomography imaging system (FMT‐4000; PerkinElmer, USA).

Furthermore, to compare the metabolic kinetics of OCY‐EVs in the brain between APP/PS1 and wild‐type (WT) mice, DiR‐labeled OCY^Young^‐EVs or OCY^Aged^‐EVs were intravenously administered (6 × 10^7^ particles in 50 µL PBS) into each mouse. The mice were sacrificed to collect tissues after 6, 24, and 48 h (*n* = 3 in each group). Fluorescent signals in these organs were detected and quantified.

Similarly, the OCY^Young^‐EVs or OCY^Aged^‐EVs were also labeled with DiL iodide (Cat. No. 40726ES10; Yeasen). After intravenous injection of DiL‐labeled OCY‐EVs, the organs were collected at indicated time points, fixed in 4% paraformaldehyde for 4 h, and embedded in Tissue‐Tek optimum cutting temperature compound (O.C.T. compound; Cat. No. 4583; Sakura, Japan) at −20 °C. Due to the particular composition of bone tissue, the femora were fixed for 24 h, decalcified in 0.5 M EDTA (pH = 7.4) at 4 °C with continuous shaking for 3 days, and then embedded in O.C.T. compound at −20 °C. Thirty‐micrometer‐thick slices were obtained using a freezing microtome (CryoStar NX50; Thermo, USA), and the fluorescence signals of DiL were observed under a fluorescence microscope (Carl Zeiss Axio Imager 2; Carl Zeiss, Germany).

### Cell Culture and Treatments

SH‐SY5Y (Cat. No. ZQ0050; Zhong Qiao Xin Zhou Biotechnology, Shanghai, China) and HT22 (Cat. No. BFN60808571; BLUEFBIO, Shanghai, China) cells were cultured in high glucose DMEM (Cat. No. C11995500BT; Gibco) supplemented with 10% FBS and 1% PS at 37 °C and 5% CO_2_ in a humidified atmosphere. SH‐SY5Y‐APPswe stable cell lines were constructed by lentiviral infection with the APPswe mutation (specials: Homo sapiens, NM_201 414.3: c.1785G>C p.K595N; c.1786A>C p.M595L), which is the most well‐known pathogenic mutation of AD.^[^ ^43^ ^]^ HT22‐APPswe cell lines were constructed by transfection of plasmids carrying the APPswe mutation. Corresponding control cell lines were transfected with empty lentivirus or plasmids. The successful constructions of APPswe cell lines were demonstrated by IF staining (Figure S7A,D, Supporting Information) and western blot (Figure S7B,C,E,F, Supporting Information) analysis of amyloid precursor protein (APP). A total of 5 × 10^4^ SH‐SY5Y‐APPswe, SH‐SY5Y, HT22‐APPswe, or HT22 cells were seeded in a 24‐well plate with 500 µL medium. After 24 h, the cell culture medium was replaced with DMEM with OCY^Young^‐EVs or OCY^Aged^‐EVs (6 × 10^7^ particles per mL). Then, the cell cultures were collected to detect A*β* levels by enzyme linked immunosorbent assay (ELISA), and the cells were analyzed for apoptosis by Hoechst 33 342 staining after 48 h.

### OCY‐EVs Uptake Assay

OCY‐EVs were labeled with PKH26 (Cat. No. MINI26; Sigma–Aldrich, Darmstadt, Germany) according to the manufacturer's instructions. After removing the redundant dye using the above‐described procedures, the labeled OCY‐EVs (6 × 10^7^ particles per mL) were incubated with SH‐SY5Y or HT22 cells at 37 °C for 12 h. Then, the treated cells were washed with PBS and fixed with 4% paraformaldehyde for 15 min. After washing with PBS, the HT22 cells were incubated with Alexa Fluor™ 488 Phalloidin (1: 200; Cat. No. A12379; Invitrogen) at 37 °C for 30 min. The stained cells on coverslips were washed twice with PBS and mounted with the commercial antifade mounting medium with DAPI (Cat. No. H‐1200‐10; Vectorlabs, California, USA). After IF staining, a fluorescence microscope was used to acquire images.

### Hoechst Staining and Apoptosis Analysis

Cells were stained with Hoechst 33 342 (Cat No.40732ES03; Yeasen) after fixation according to the manufacture's protocol. A fluorescence microscope was used to acquire images. As the nuclei of apoptotic cells were concentrated,^[^ ^44^ ^]^ apoptotic cells showed a high fluorescence intensity by Hoechst staining and were considered Hoechst‐positive cells. The percentage of Hoechst‐positive cells was analyzed using ImageJ software (NIH).

### Animal Experiments

Animal care and experimental procedures were approved by the Ethical Review Board at Xiangya Hospital of Central South University (2019030501). APP/PS1 mice (Stock No. 34829‐JAX) and *Dmp1^Cre^* mice (Stock No. 02 3047) were imported from Jackson Laboratory. C57BL/6 mice were obtained from Hunan SJA Laboratory Animal Co. Ltd.

EVs reporter (*Cd63^fl/+^*) mice and osteocyte specific targeting (*Sost^CreERT2^*) mice were established using homologous recombination CRISPR/Cas9 technology. Cas9 mRNA and gRNA were obtained by in vitro transcription. For *Cd63^fl/+^* mice, a homologous recombination vector (donor vector) contained a 3.4 kb 5′ homologous arm, *loxp‐cc‐mCherry‐polyA‐loxp‐cc‐eGFP*, and a 4.0‐kb 3′ homologous arm. For *Sost^CreERT2^* mice, a homologous recombination vector (donor vector) contained a 3.0 kb 5′ homologous arm, *CreERT2‐Wpre‐pA*, and 3.0 kb 3′ homologous arm. Cas9 mRNA, gRNA, and donor vector were microinjected into the fertilized eggs of C57BL/6J mice to obtain F0 generation mice. Homologous recombinant F0 generation mice were identified by long fragment PCR. To activate CreERT2, 100 mg kg^−1^ tamoxifen (Cat. No. ST1681; Beyotime) was administered once a day for five consecutive days by intraperitoneal manner. The genetic identification results of *Sost^CreERT2^;CD63^fl/+^* mice, *DMP1^Cre^;CD63^fl/+^* mice, and APP/PS1 mice are shown in Figure S2A–D in the Supporting Information.

Four‐month‐old early stage AD model mice were intravenously administered by OCY^Young^‐EVs, OCY^Aged^‐EVs, or solvent weekly based on the weight of the mouse (2.4 × 10^6^ particles per gram, 1.2 × 10^6^ particles per µL) for two months. Six‐month‐old later stage AD model mice were intravenously administered by OCY^Young^‐EVs, OCY^Aged^‐EVs, or solvent (4.8 × 10^6^ particles per gram, 1.2 × 10^6^ particles per µL) weekly for two months. Behavior tests were performed after the interventions. Then, the brain and femora were harvested for downstream measurements.

An adenovirus carrying small hairpin RNA targeting Rab27a (*Ad‐Rab27a*‐shRNA) was constructed to inhibit the EVs secretion from the bone. AD model mice were constructed by injecting A*β*40 into the hippocampus. Eight‐week‐old C57BL/6 male mice were randomly divided into four groups: Ad‐Mock/Solvent, Ad‐Mock/A*β*40, *Ad‐Rab27a*‐shRNA/Solvent, and *Ad‐Rab27a*‐shRNA/A*β*40. The mice were intramedullary injected by 5 µL (5 × 10^10^ IFU mL^−1^) of *Ad‐Rab27a*‐shRNA or Ad‐Mock at the bilateral femur under anesthesia. After seven days, the mice were placed in a stereotactic device and bilaterally administered 3 µL A*β*40 (1 µg µL^−1^) or solvent into the hippocampus under anesthesia.^[^ ^45^ ^]^ The A*β*1‐40 monomer solution (Cat. No. 2027‐2‐20; ChinaPeptides, Shanghai, China) was incubated at 37 °C for three days before use. The injection location of the hippocampus was −2.0 mm posterior to the bregma, ±1.3 mm lateral to the sagittal suture, and 1.7–1.8 mm in depth (Figure S10, Supporting Information). Behavior tests were performed two weeks later. After the test, the brain and femora were harvested for downstream measurements.

### Morris Water Maze

The apparatus consisted of a white circular pool (130 cm diameter, 51 cm high) filled with water (30 cm depth at 22 °C) and divided into four quadrants. An invisible circular platform (10 cm in diameter) was placed in the middle of one of the quadrants and submerged 1 cm below the surface. A distal clue was also placed for mice to locate the platform. A commercial animal video‐tracking analysis system (AVTAS ver4.0; Wuhan, China) was used for the Morris Water Maze (MWM). Animals were first subjected to water maze training and four trials from four different fixed start positions each day until most of them arrived at the platform quickly. Each trial lasted 60 s or until the animal found the platform. All animals were left on the platform for 10 s (including those who failed to locate it). The interval between each training was 1 h so that the mice can regain their physical strength. After the last training session, the mice were subjected to the probe trail for 60 s. The platform was removed from the pool in the probe trial, and the time spent in the platform quadrant and the platform crossing time were measured.

### Y‐Maze

The apparatus was a symmetrical Y‐Maze; each arm measured 50 × 10 cm with 20‐cm‐high sidewalls and was labeled with different shapes. Mice were placed in one arm (start arm) of the maze with a closed arm and allowed to explore freely for 10 min. The unexplored arm is named the novel arm, and the remaining arm is named the familiar arm. After 2 h, the mice were placed in the start arm (all three arms were open) and the maze was explored for 5 min. The times in each arm were recorded.

### Object Location Task and Novel Object Recognition Task

The apparatus consisted of a square area (30 × 30 cm) with 30‐cm‐high sidewalls. Mice were placed into the testing room to acclimate for at least 30 min and then allowed to explore the arenas freely for 10 min. The arenas were cleaned to minimize olfactory cues before the next use. For the training trial, two different objects were secured to the open field at 6 × 6 cm away from their respective walls. After a duration of 20 min, the animals were placed facing the walls in the release corner and allowed to investigate the arena and objects freely for 10 min. For the object location task (OLT), one object was moved to a new location 6 × 6 cm away from the walls and not the release corner. The animals were placed in the same way and allowed to investigate the arena and objects freely for 10 min after 20 min rest. For the novel object recognition task (NORT), the stationary object in the OLT was replaced with a novel object, while the moving object from the OLT was now the familiar object. Twenty minutes later, the animals were placed for the third time and allowed to investigate the arena and objects freely for 10 min. The investigation times for each mouse with each object were recorded and analyzed.

### IF Staining

For IF staining, brain tissues were fixed in 4% paraformaldehyde for 24 h. Subsequently, the samples were dehydrated in 30% glucose with 1% polyvinyl pyrrolidone (PVP; Cat. No. 9003‐39‐8; Sigma–Aldrich) at 4 °C for ≈24 h until the tissues sank to the bottom, and they were then embedded in O.C.T. compound at −20 °C. Ten‐micrometer‐thick slices were incubated with different antibodies: SOST (1: 500; Cat. No. AF1589; R&D, Minnesota, USA), GFP (1: 50 000; Cat. No. A6290; Abcam, Cambridge, England), 6E10 (1: 500; Cat. No. 803 001; Biolegend, San Diego, CA, USA), Iba1 (1: 500; Cat. No. ab178847; Abcam), Synaptophysin (1: 500; Cat. No. MA5‐14532; Invitrogen), or NeuN (1: 500; Cat. No. MAB377; Millipore). Appropriate secondary antibodies (Abcam) were used, followed by incubation with DAPI. A total of six sections per brain containing the hippocampus and cortex were stained. Fluorescence microscopy and Pannoramic Scanner (Pannoramic MIDI; 3D HISTECH, Hungary) were used to acquire images.

### Image Quantification

After IF staining, confocal images were captured, and the mean intensity of fluorescence, number of immunoreactive cells, and size of the plaques were quantified. To quantify 6E10 in the mouse brain, six sections (including the hippocampus) were scanned using a Pannoramic Scanner. Images were then analyzed by Image Pro‐Plus (MEDIA CYBERNETICS), and the background was subtracted by the software for fluorescence images before quantification. For A*β* plaques, the number of plaques, the size of plaques, and the intensity of 6E10 in six brain sections containing the hippocampus and cortex of each mouse were measured. For A*β* plaque‐associated Iba1 intensity quantification, Iba1 intensities were counted within the 100‐µm radius of the 6E10‐positive plaques. At least 100 plaques were counted in each group, and Iba1 intensities were normalized to the plaques. For synapse studies, synaptophysin (Syp) intensities in the cortex and hippocampus were quantified by Image Pro‐Plus. Because of the great variation in synaptic puncta densities by brain region, care was taken to image the same areas consistently across all samples. For NeuN‐positive cell quantification, six images from the cortex or the scanned images of the hippocampus of each mouse were counted manually. All images were captured and analyzed blindly using coded slides.

### ELISA

The concentrations of A*β*42 and A*β*40 were measured using commercial ELISA kits (Cat. No. DAB142, DAB140B; R&D). All procedures were performed in accordance with the manufacturer's instructions.

### Western Blot

Cells or brain tissues were collected and resuspended in modified RIPA lysis buffer (Cat. No. P0013B; Beyotime, Shanghai, China) containing protease and phosphatase inhibitor mixture. The suspensions were lysed on ice for 30 min, sonicated, and then centrifuged at 13 000 rpm for 10 min at 4 °C. Protein concentrations were estimated using the BCA kit (Cat. No. 70‐PQ0012; Multi Sciences). Lysates were separated on 8%, 10%, 12%, and 15% SDS–polyacrylamide electrophoresis (SDS–PAGE) gels (Cat. No. P1200; Solarbio) or 15% Tris‐Tricine‐SDS–PAGE gels (Cat. No. P1320; Solarbio). After the separation, proteins were transferred to a PVDF membrane (Cat. No. IPVH00010, ISEQ00010; Millipore), and nonspecific binding sites were blocked using commercial western blocking buffer (Cat. No. SW3010; Solarbio) followed by antibody incubation: TSG101 (1: 1000; Cat. No. sc‐7964; Santa Cruz, Dallas, Texas, USA), Flotillin‐1 (1: 500; Cat. No. sc‐74566; Santa Cruz), DMP1 (1: 1000; Cat. No. NBP1‐45525; NOVUS, Colorado, USA), SOST (1: 500; Cat. No. AF1589; R&D), P21 (1: 1000; Cat. No. ab188224; Abcam), P16 (1: 2000; Cat. No. ab211542; Abcam), 6E10 (1: 1000; Cat. No. 803 001; Covance), APP (1: 1000; Cat. No. ab32136; Abcam), or *β*‐actin (1: 1000; Cat. No. O10313; TransGen Biotech, Beijing, China). Secondary HRP–conjugated antibodies (1: 5000; Solarbio) were used. Western blot images were captured using the ChemiDoc XRS+ with Image Lab Software (Bio–Rad, California, USA). The western blot bands were quantified using ImageJ software.

### qRT–PCR

Bone and bone marrow samples were freshly collected. Total RNA was extracted using the *TransZol* Up Plus RNA Kit (Cat. No. ER501‐01; Transgen) and cDNA was synthesized from 1 µg of total RNA using the NovoSceipt Plus All‐in‐one 1^st^ Strand cDNA synthesis kit (Cat. No. E047; Novoprotein, Shanghai, China). The cDNA was then amplified with 2× SYBR Green qPCR Master Mix (Cat. No. B21202; Bimake, Texas, USA) in an FTC‐3000 real‐time PCR system (Funglyn Biotech Inc, Toronto, Canada). The relative standard curve method (2^−ΔΔCt^) was used to determine the relative gene expression and *Gapdh* was used as a housekeeping gene for internal normalization. The PCR primers used in this study were as follows: mouse‐*Rab27a*: forward, 5″‐TGGAAGGGAGTACCTAAGGGAT‐3″, and reverse, 5″‐CTCAGCCAGGGTTGATGAGATA‐3″ ; mouse‐*Gapdh*: 5″‐CACCATGGAGAAGGCCGGGG‐3″, and reverse, 5″‐GACGGACACATTGGGGGTAG‐3″.

### Proteomic Analysis

OCY‐EVs samples (OCY^Young^‐EVs and OCY^Aged^‐EVs, *n* = 3 for each) were prepared as described previously,^[^ ^46^ ^]^ and processed for label‐free quantitative proteomic analysis by Jingjie PTM BioLab (Hangzhou, China). Differentially enriched proteins were identified (OCY^Young^‐EVs/OCY^Aged^‐EVs > 1.5 or < 0.67, *P* < 0.05). Enrichment of functions and signaling pathway analysis were performed based on Gene Ontology (GO) and the Kyoto Encyclopedia of Genes and Genomes (KEGG) database at the David site (https://david.ncifcrf.gov).^[^ ^47^ ^]^ For functional enrichment analysis, differentially enriched proteins (OCY^Young^‐EVs/OCY^Aged^‐EVs > 1.5) were mapped to terms in the GO databases, and then significantly enriched GO terms were searched using *P* < 0.05 as the threshold. GO term analysis was classified into three subgroups, namely biological process (BP), cellular component (CC), and molecular function (MF). The differentially enriched proteins (OCY^Young^‐EVs/OCY^Aged^‐EVs > 1.5) were also mapped to the KEGG database and searched for significantly enriched KEGG pathways at the *P* < 0.05 level.

### Statistical Analysis

Continuous variables are expressed as mean ± SD. The sample size (*n*) for each statistical analysis was detailed in figure legends. The Gender, T‐Score Classification, and BMD Classification data of clinical subjects were analyzed by the Chi‐Square Test. Spearman Correlation Analysis was adopted between the T‐Score and MMSE. The unpaired, two‐tailed Student's *t*‐test was used to analyze the differences between two groups. The significance of multiple‐group comparisons was performed by one‐way analysis of variance (ANOVA) or Kruskal–Wallis test with Bonferroni post hoc correction. For all experiments, *P* < 0.05 was considered as significant, and was represented by “*/#”; *P* < 0.01, *P* < 0.001, and *P* < 0.0001 were represented by “**/##”, “***/###”, and “****/####”. GraphPad Prism software (Version 8.0) and SPSS (Version 23.0) were used for the above statistical analyses.

## Conflict of Interest

The authors declare no conflict of interest.

## Author Contributions

Y.‐L.J. and Z.‐X.W. contributed equally to this work. L.S., H.X., Y.‐L.J., and Z.‐X.W. conceived the project and designed the experiments. Y.‐L.J., X.‐X.L., M.‐D.W., Y.‐W.L., Y.‐Y.W., T.‐F.W., and Y.Z. performed the experiments. L.S., H.X., B.J., X.‐X.L., Z.‐W.L., C.‐G.H., Y.‐J.T., L.W., Y.‐F.Z., S.‐S.R., J.C., and Z.‐Z.L. contributed to the experimental technical consultations. Y.‐L.J. and Z.‐X.W. contributed to the data acquisition and analysis. L.S., H.X., Y.‐L.J., and Z.‐X.W. wrote the manuscript. All authors reviewed and revised the manuscript.

## Supporting information

Supporting Information

Supporting Information

## Data Availability

The data that support the findings of this study are available from the corresponding author upon reasonable request.
