# Supplementary material for: The Protective Effects of Osteocyte‐Derived Extracellular Vesicles Against Alzheimer's Disease Diminished with Aging
Source: Adv Sci (Weinh). 2022 May 4;9(17):2105316. doi: 10.1002/advs.202105316 (PMC9189667; doi:10.1002/advs.202105316)
Supplement: Supplementary file 1 — Supporting Information [file ADVS-9-2105316-s001.pdf]

ADVANCED SCIENCE
Open Access

# Supporting Information

for *Adv. Sci.*, DOI 10.1002/advs.202105316

The Protective Effects of Osteocyte-Derived Extracellular Vesicles Against Alzheimer's Disease Diminished with Aging

*Ya-Ling Jiang, Zhen-Xing Wang, Xi-Xi Liu, Mei-Dan Wan, Yi-Wei Liu, Bin Jiao, Xin-Xin Liao, Zhong-Wei Luo, Yi-Yi Wang, Chun-Gu Hong, Yi-Juan Tan, Ling Weng, Ya-Fang Zhou, Shan-Shan Rao, Jia Cao, Zheng-Zhao Liu, Teng-Fei Wan, Yuan Zhu, Hui Xie* and Lu Shen**

Supporting Information

# The Protective Effects of Osteocyte-derived Extracellular Vesicles against Alzheimer's Disease Diminished with Aging

Ya-Ling Jiang#, Zhen-Xing Wang#, Xi-Xi Liu, Mei-Dan Wan, Yi-Wei Liu, Bin Jiao, Xin-Xin Liao, Zhong-Wei Luo, Yi-Yi Wang, Chun-Gu Hong, Yi-Juan Tan, Ling Weng, Ya-Fang Zhou, Shan-Shan Rao, Jia Cao, Zheng-Zhao Liu, Teng-Fei Wan, Yuan Zhu, Hui Xie*, Lu Shen*

## Supplementary method

***μCT analysis*:** Femora were fixed overnight in 4% paraformaldehyde and analyzed by high-resolution *μ*CT (VIVACT 80; SCANCO Medical AG, Switzerland) as described in the previous studies.[1] The scanner was set at a current of 200 μA and a voltage of 70 kV, respectively. The isotropic voxel size, X-ray tube potential, and integration time were set at 11.4 × 11.4 × 11.4 $\mu m^3$, 55 kV, and 400 ms, respectively. The image reconstruction software (NRecon), data analysis software (CTAn v1.11), and 3D model visualization software (*μ*CTVol v2.2) were applied to analyze the parameters of the distal femoral metaphyseal trabecular bone and the diaphyseal cortical bone. The region of interest (ROI) selected for trabecular bone analysis was started from 0.3 mm proximal to the distal growth plate and extended proximally for 5% of femoral length, which excluded the growth plate and primary spongiosa. The fixed threshold values were set to 60 as the lower limit and 255 as the upper limit to distinguish mineralized bone from other tissues. Percent bone volume of trabecular bone (Tb. BV/TV), Trabecular number (Tb. N), Trabecular separation (Tb. Sp), and Trabecular thickness (Tb. Th) were measured. For cortical bone, the ROI selected for scanning was started from 40% of femoral length proximal to the distal growth plate and extended proximally for 10% of femoral length, by which percent bone volume of cortical bone (Ct. BV/TV), periosteal perimeter (P. Pm), endocortical perimeter (E. Pm) and crossectional thickness (Ct. Th) were calculated.

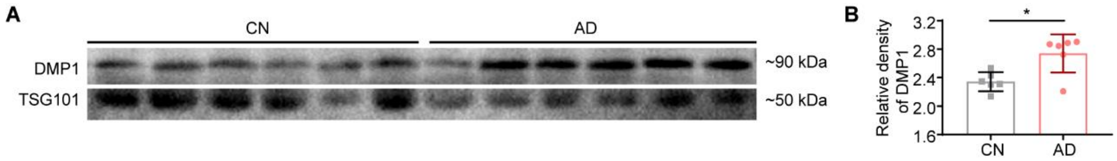


**Figure S1. Representative Plasma EVs from CN and AD Subjects in One Gel.** A) Western blot images of DMP1 and TSG101 in plasma EVs from gender- and age-matched CN subjects and AD patients. B) Quantification of DMP1 intensity in A). Data were presented as mean ± SD. For panel (B): unpaired, two-tailed Student's *t*-test. * $P < 0.05$.

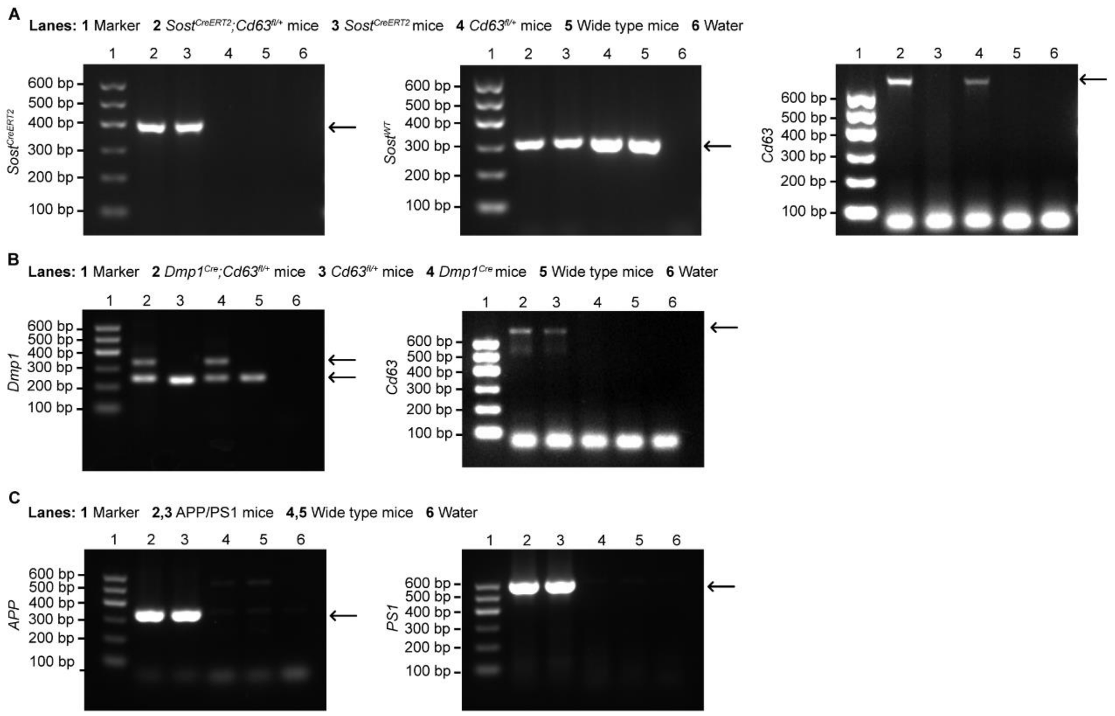


D

| Gene | Primer sequence | | Annealing temperature | Size |
|---|---|---|---|---|
| *Sost*$^{CreERT2}$ | Forward | 5'-TGAGGAAACATGGGACCAGC-3' | 60 ℃ | 390 bp (Mut) |
| | Reverse | 5'-CGCGCGCCTGAAGATATAGA3' | | |
| *Sost* | Forward | 5'-TGCTCCAGCCCGTCCTTATTG-3' | 60 ℃ | 286 bp (WT) |
| | Reverse | 5'-CGCCCGGTTCATGGTCTGGT-3' | | |
| *Dmp1*$^{Cre}$ | Forward | 5'-CACGTCCTCTCACTTCTCACG-3' | 60 ℃ | 341 bp (Mut) |
| | Reverse | 5'-CTTTGACAGTGTCTTATCCAATAGCC-3' | | 240 bp (WT) |
| *Cd63* | Forward | 5'-TGAGCAAGGGCGAGGAGGATAACA-3' | 63 ℃ | 689 bp |
| | Reverse | 5'-CATGCCGCCGGTGGAGTGG-3' | | |
| *APP* | Forward | 5'-GACTGACCACTCGACCAGGTTCTG-3' | 65 ℃ | 350 bp |
| | Reverse | 5'-CTTGTAAGTTGGATTCTCATATCCG-3' | | |
| *PSEN1* | Forward | 5'-AATAGAGAACGGCAGGAGCA-3' | 60 ℃ | 608 bp |
| | Reverse | 5'-GCCATGAGGGCACTAATCAT-3' | | |

**Figure S2. Identifications of Transgenic Mice.** A-B) Genotyping identification of *Sost*$^{CreERT2}$, *Dmp1*$^{Cre}$, and *Cd63*$^{fl/+}$ for indicated mice. C) Genotyping identification of APP/PS1 mice. D) Primers in (A-C).

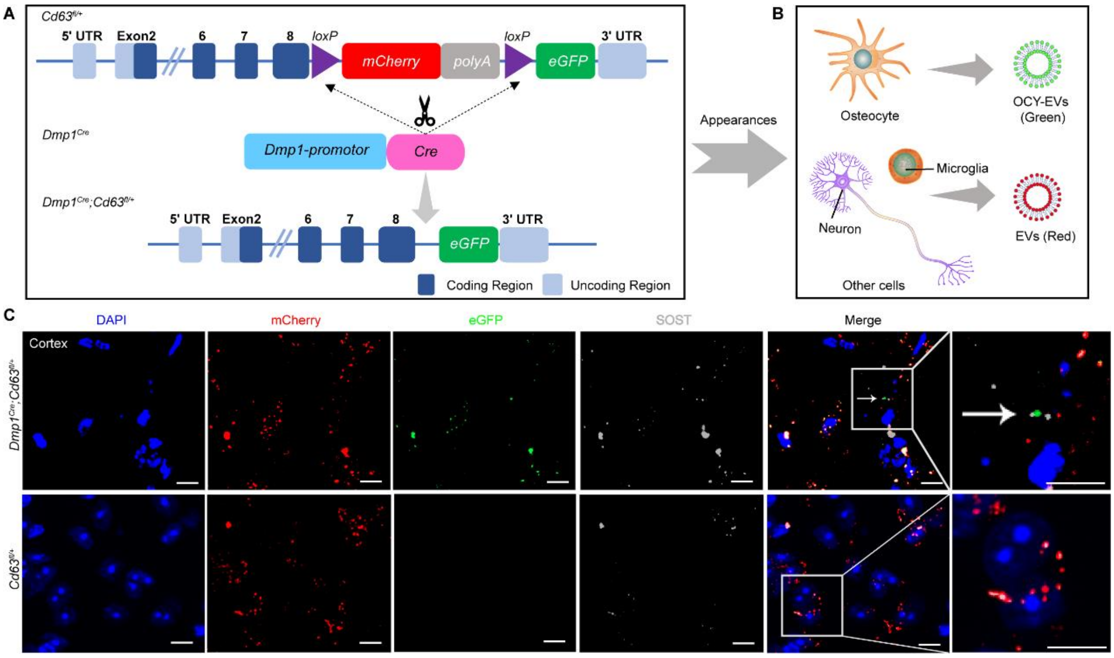


**Figure S3. OCY-EVs were Transported to the Brain under Physiological Conditions in *Dmp1$^{Cre}$;Cd63$^{fl/+}$* Mice.** A) Schematic diagram of the construction of *Dmp1$^{Cre}$;Cd63$^{fl/+}$* mice model and B) its fluorescent appearances of EVs. C) Representative IF images of brain tissue sections of *Dmp1$^{Cre}$;Cd63$^{fl/+}$* mice and *Cd63$^{fl/+}$* littermate control mice. DAPI (blue), mCherry (red), eGFP (green), and SOST (gray). The merged photos were overlaid on the right side, and the white square indicated the amplification regions. Scale bar: 10 μm.

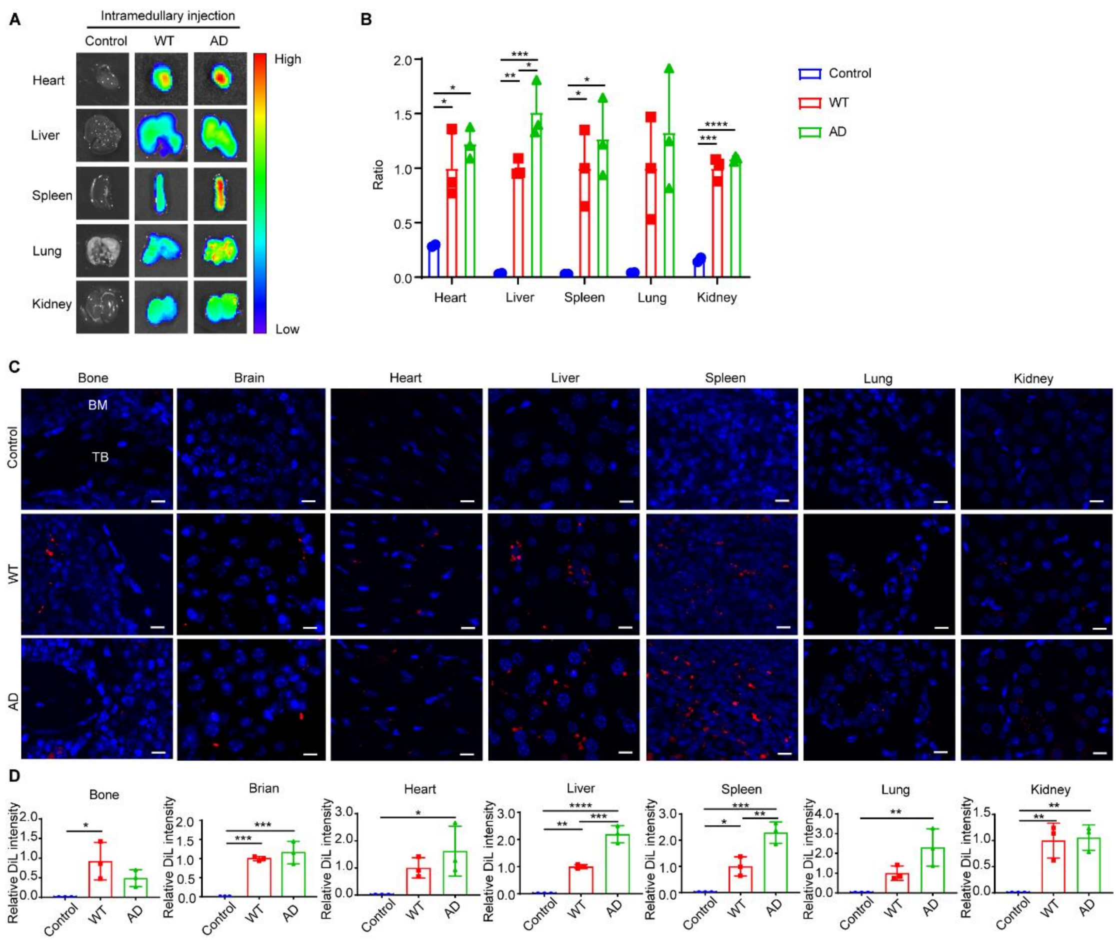

**Figure S4. Intramedullary Administered OCY-EVs Transported from Bone Tissue to Extraskeletal (nonbone) Tissues under Pathological Conditions.** A) Representative *ex vivo* fluorescent images and B) quantification of the fluorescent signals in heart, liver, spleen, lung, and kidney from WT and APP/PS1 mice treated with intra-bone marrow administered DiR-labeled OCY-EVs or solvent for 24 h. n = 3 per group. C) Representative fluorescent images and D) quantification of the relative DiL intensity in bone, brain, heart, liver, spleen, lung, and kidney sections of WT and APP/PS1 mice treated with intra-bone marrow administered DiL-labeled OCY-EVs or solvent for 24 h. The nucleus was stained by DAPI (blue). TB: trabecular bone; BM: bone marrow. n = 3 per group. Scale: 10 μm. Data were presented as mean ± SD. For panel (B, D): one-way ANOVA or the Kruskal–Wallis test with Bonferroni post hoc correction. * $P < 0.05$, ** $P < 0.01$, *** $P < 0.001$, **** $P < 0.0001$.

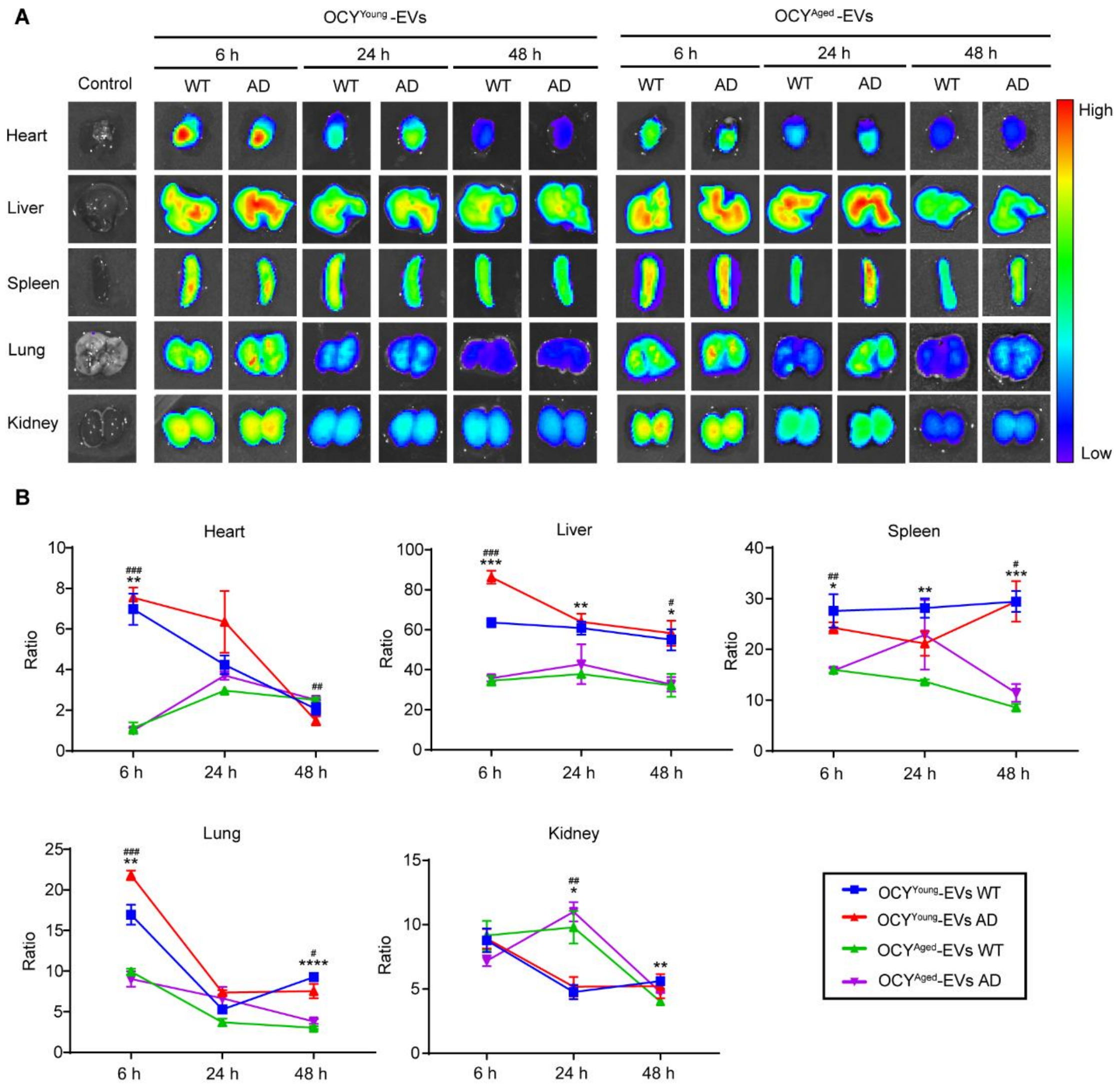

**Figure S5. Tissue Distribution Dynamics of OCY$^{2M}$-EVs and OCY$^{16M}$-EVs in WT and APP/PS1 Mice.** Representative *ex vivo* fluorescent images A) and quantified fluorescent signals B) of the heart, liver, spleen, lung, and kidney from WT and APP/PS1 mice intravenously treated with DiR-labeled OCY$^{Young}$-EVs, DiR-labeled OCY$^{Aged}$-EVs, or solvent for 6 h, 24 h, and 48 h. n = 3 per group. * OCY$^{Young}$-EVs WT (blue line) *versus* OCY$^{Aged}$-EVs WT (green line), # OCY$^{Young}$-EVs AD (red line) *versus* OCY$^{Aged}$-EVs AD (purple line). Data were presented as mean ± SD. For panel (B): unpaired, two-tailed Student's *t*-test. */# $P < 0.05$, **/## $P < 0.01$, ***/### $P < 0.001$, ****/#### $P < 0.0001$.

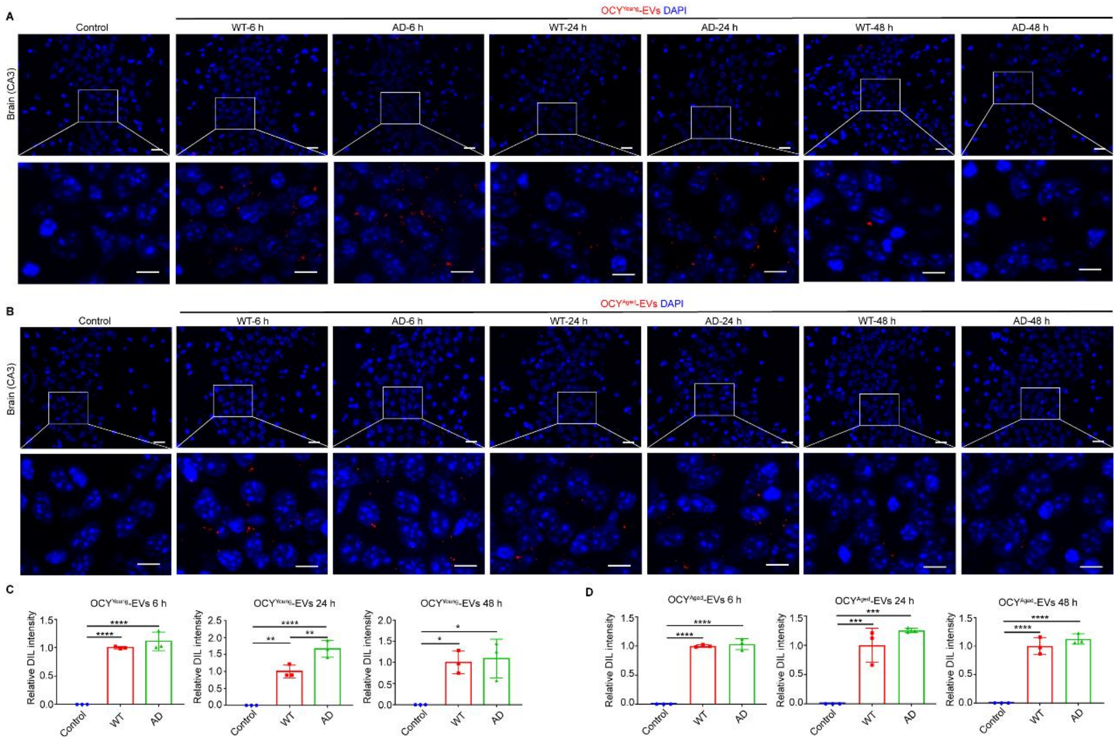


**Figure S6. OCY$^{Young}$-EVs might Stay Longer in the Brain of APP/PS1 Mice.** A-B) Representative fluorescent images and C-D) quantified fluorescent signals in brain sections of WT and APP/PS1 mice intravenously treated with DiL-labeled OCY$^{Young}$-EVs (A and C), DiL-labeled OCY$^{Aged}$-EVs (B and D), or solvent for 6 h, 24 h, and 48 h. The nucleus was stained by DAPI (blue). The white square indicated the amplification regions. Scale: 20 μm (brain CA3), 10 μm (magnified images). n = 3 per group. Data were presented as mean ± SD. For panel (C-D): one-way ANOVA or the Kruskal–Wallis test with Bonferroni post hoc correction. * $P < 0.05$, ** $P < 0.01$, *** $P < 0.001$, **** $P < 0.0001$.

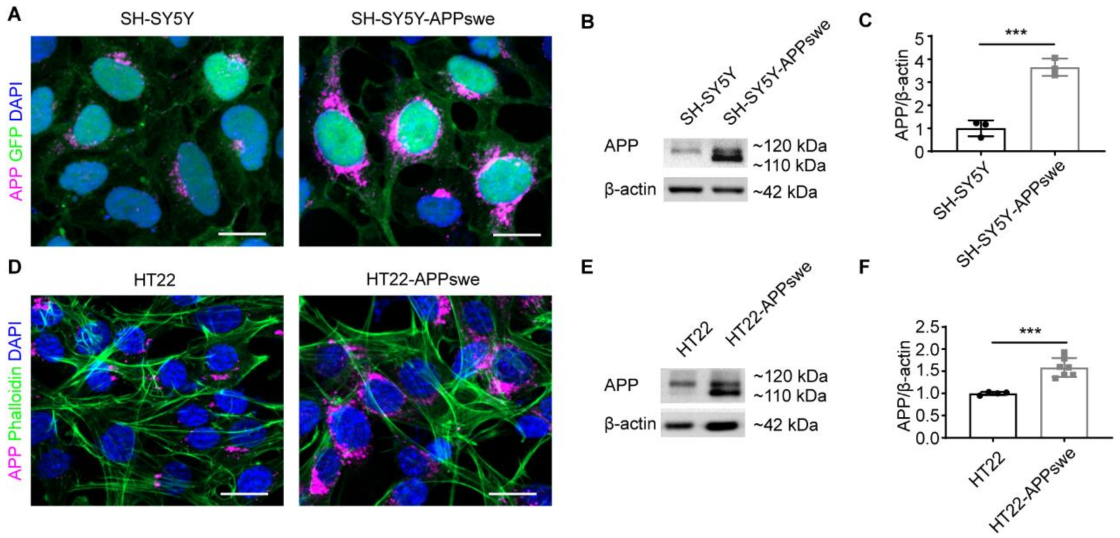


**Figure S7. Identifications of AD Model Cells.** A) Representative IF images for APP (purple), GFP (green), and DAPI (blue) of SH-SY5Y and SH-SY5Y-APPswe cells. scale bar: 20 μm. B) Representative western blot images and C) quantification of APP expressions in SH-SY5Y and SH-SY5Y-APPswe. n = 3 per group. D) Representative IF images for APP (purple), Phalloidin (green), and DAPI (blue) of HT22 and HT22-APPswe cells. scale bar: 20 μm. E) Representative western blot images and F) quantification of APP expressions in HT22 and HT22-APPswe cells. n = 6 per group. Data were presented as mean ± SD. For panel (C) and (F): unpaired, two-tailed Student's *t*-test. *** $P < 0.001$.

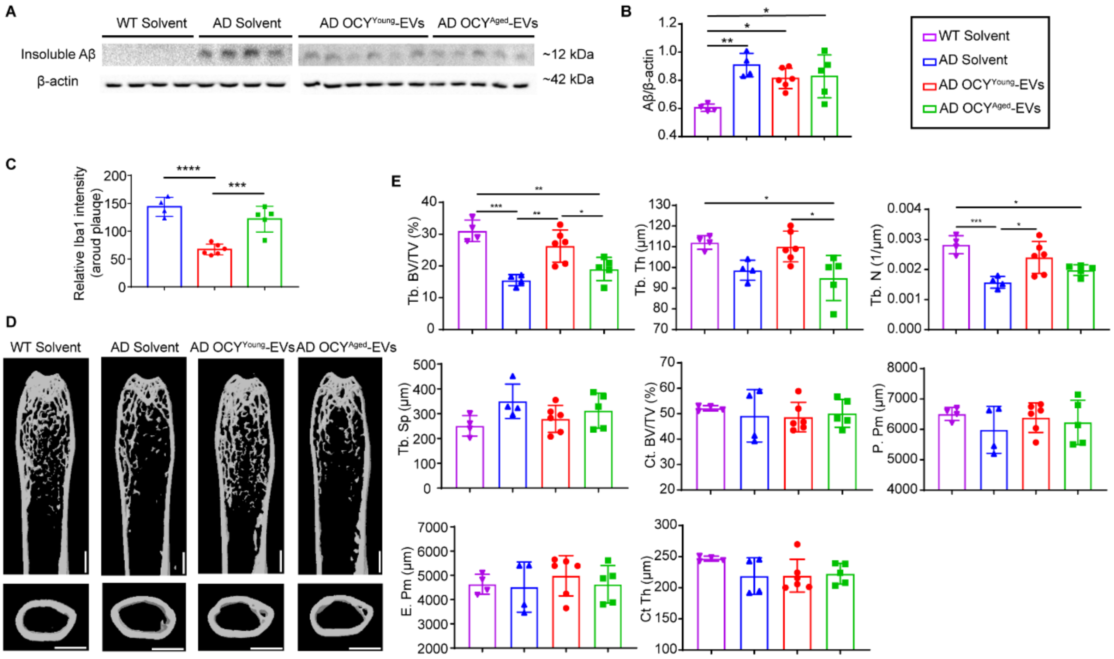

**Figure S8. Insoluble Aβ and Bone Quality in the Early Stage AD Model Mice.** A) Western blot images of the insoluble Aβ by 6E10 antibody and B) quantification of 6E10 intensity of brain samples from different treated mice. C) Quantification of the relative Iba1 intensity in the brain sections of Figure 4L. D) Representative μCT reconstruction images and E) quantification of bone microarchitecture parameters in the femurs from different treated mice. Scale bars: 1 mm. WT group, n = 4; solvent-treated APP/PS1 group, n = 4; $OCY^{Young}$-EV-treated APP/PS1 group, n = 6; $OCY^{Aged}$-EV-treated group, n = 5. Data were presented as mean ± SD. For panel (B), (C), and (E): one-way ANOVA or the Kruskal–Wallis test with Bonferroni post hoc correction. * $P < 0.05$, ** $P < 0.01$, *** $P < 0.001$.

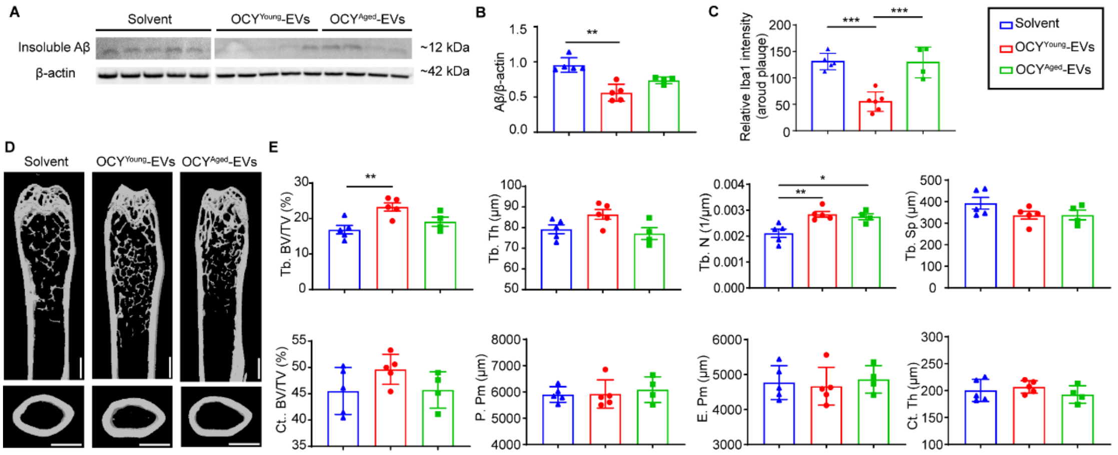


**Figure S9. Insoluble Aβ and Bone Quality in the Later Stage AD Model Mice.** A) Western blot images of the insoluble Aβ by 6E10 antibody and B) quantification of 6E10 intensity of brain samples from different treated mice. C) Quantification of the relative Iba1 intensity in the brain sections of Figure 5L. D) Representative μCT reconstruction images and E) quantification of bone microarchitecture parameters in the femurs from different treated mice. Scale bars: 1 mm. Solvent-treated APP/PS1 group, n = 5; OCY$^{Young}$-EV-treated APP/PS1 group, n = 5; OCY$^{Aged}$-EV-treated group, n = 4. Data were presented as mean ± SD. For panel (B), (C), and (E): one-way ANOVA or the Kruskal–Wallis test with Bonferroni post hoc correction. * $P < 0.05$, and ** $P < 0.01$.

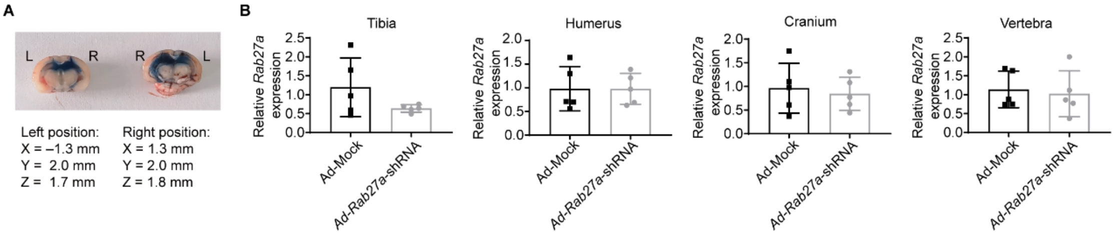


**Figure S10. The Administration of Aβ40 and *Ad-Rab27a*-shRNA.** A) Cerebral coronal image of mice treated with bilateral hippocampus administrated of trypan blue. B) qRT-PCR analysis of *Rab27a* mRNA expression levels in the tibia, humerus, cranium, and vertebra samples from *Ad-Rab27a*-shRNA or Ad-Mock treated mice. n = 5 per group. Data were presented as mean ± SD. For panel (B): unpaired, two-tailed Student's *t*-test.

**Reference**

[1] a) J. H. Liu, C. Y. Chen, Z. Z. Liu, Z. W. Luo, S. S. Rao, L. Jin, T. F. Wan, T. Yue, Y. J. Tan, H. Yin, F. Yang, F. Y. Huang, J. Guo, Y. Y. Wang, K. Xia, J. Cao, Z. X. Wang, C. G. Hong, M. J. Luo, X. K. Hu, Y. W. Liu, W. Du, J. Luo, Y. Hu, Y. Zhang, J. Huang, H. M. Li, B. Wu, H. M. Liu, T. H. Chen, Y. X. Qian, Y. Y. Li, S. K. Feng, Y. Chen, L. Y. Qi, R. Xu, S. Y. Tang, H. Xie, *Adv Sci* **2021**, 8, 2004831; b) C. Y. Chen, S. S. Rao, Y. J. Tan, M. J. Luo, X. K. Hu, H. Yin, J. Huang, Y. Hu, Z. W. Luo, Z. Z. Liu, Z. X. Wang, J. Cao, Y. W. Liu, H. M. Li, Y. Chen, W. Du, J. H. Liu, Y. Zhang, T. H. Chen, H. M. Liu, B. Wu, T. Yue, Y. Y. Wang, K. Xia, P. F. Lei, S. Y. Tang, H. Xie, *Bone Res* **2019**, 7, 18.
